# Kinetic Microscale Thermophoresis for Simultaneous Measurement of Binding Affinity and Kinetics

**DOI:** 10.1002/anie.202101261

**Published:** 2021-05-11

**Authors:** Julian A. C. Stein, Alan Ianeselli, Dieter Braun

**Affiliations:** ^1^ Systems Biophysics Department of Physics Ludwig-Maximilians-Universität München and Center for NanoScience Amalienstasse 54 80799 München Germany

**Keywords:** binding kinetics, DNA hybridization, DNA thermodynamics, kinetic rates, microscale thermophoresis

## Abstract

Microscale thermophoresis (MST) is a versatile technique to measure binding affinities of binder–ligand systems, based on the directional movement of molecules in a temperature gradient. We extended MST to measure binding kinetics as well as binding affinity in a single experiment by increasing the thermal dissipation of the sample. The kinetic relaxation fingerprints were derived from the fluorescence changes during thermodynamic re‐equilibration of the sample after local heating. Using this method, we measured DNA hybridization on‐rates and off‐rates in the range 10^4^–10^6^ m ^−1^ s^−1^ and 10^−4^–10^−1^ s^−1^, respectively. We observed the expected exponential dependence of the DNA hybridization off‐rates on salt concentration, strand length and inverse temperature. The measured on‐rates showed a linear dependence on salt concentration and weak dependence on strand length and temperature. For biomolecular interactions with large enthalpic contributions, the kinetic MST technique offers a robust, cost‐effective and immobilization‐free determination of kinetic rates and binding affinity simultaneously, even in crowded solutions.

## Graphical abstract

The extension of microscale thermophoresis (MST) to perform temperature jumps by increasing the heat dissipation of the sample allows for quantification of binding affinities together with the kinetic rates. Relaxation kinetics for DNA hybridization were measured with high fidelity at different temperatures and various experimental conditions. The results shed light on the hybridization mechanism of DNA and confirmed determinants of DNA binding.

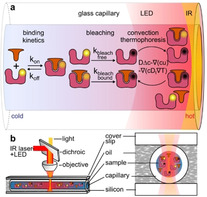

## Introduction

The dissociation constant *K*_d_=*k*_off_/*k*_on_ characterizes the binding affinity of a binder–ligand system and has been extensively studied in many research fields.[^1^, ^2^, ^3^, ^4^] *K*_d_ is usually determined by the analysis of equilibrated states of binder–ligand systems. On the other hand, the determination of the underlying kinetic association and dissociation rates *k*_on_ (on‐rate) and *k*_off_ (off‐rate) usually requires the time‐resolved measurement of the transition of the system from a non‐equilibrium state towards equilibrium.^[5]^ The necessary deflection from equilibrium can be introduced to the system either by rapid mixing of the reactants^[6]^ or by rapid temperature jumps.^[7]^ During this transition, the change in concentration of bound and unbound molecules is governed by the kinetic rates.^[8]^ The kinetic rates provide a more thorough understanding of the binding process, because they characterize the binding (on‐rate dependent), the dissociation (off‐rate dependent) of the complex and the timescales of the binding process, as well as its stability. However, due to the difficulty of their measurement, kinetic rates have not received as much attention as the dissociation constant.[^8^, ^9^]

The measurement of kinetics by rapid mixing of reactants often requires immobilization of one of the reactants. The free ligand is then added to the mixture containing the immobilized binder for a defined period and the subsequent binding is recorded, for example, by surface‐plasmon resonance measurements (SPR),^[10]^ nanotube biosensors^[9]^ and biolayer interferometry (BLI).^[11]^ SPR and BLI offer label‐free detection and real‐time data acquisition and are independent of temperature‐related characteristics. Immobilization‐based methods that apply electric potentials to expose the ligand and the binder are useful for studying systems such as aptamer–analyte complexes.^[12]^ However, immobilization might alter the chemical and physical properties of biomolecules^[13]^ leading to modified binding properties or even loss of binding in extreme cases,^[14]^ for example, the binding site could be inaccessible due to random orientation of the molecule attached to the surface.^[15]^ It was reported for SPR that the binding affinity could be overestimated due to underestimated off‐rates.[^1^, ^16^] To conclude, the immobilization techniques are suitable for studies of interactions near or on surfaces, but not ideal for studying in‐solution interactions.

Many physiological interactions take place in crowded solutions. Experimental methods which allow determination of kinetic rates under such conditions and without immobilization include fluorescence anisotropy (FA),^[17]^ fluorescence correlation spectroscopy (FCS),^[18]^ Förster Resonance Transfer (FRET)^[19]^ and fluorescence quenching (coupled to stopped‐flow technique)^[6]^ among others.^[20]^ Even though all these are excellent options for determining the kinetic rates, they often suffer from prohibitive costs, time‐consuming sample preparation steps and extensive data analysis.

**Figure 1:**
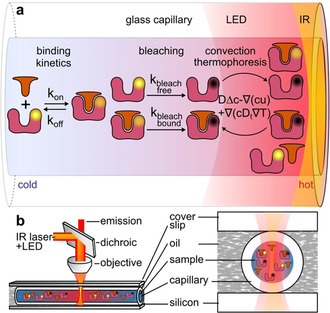
Kinetic microscale thermophoresis setup. a) Molecular interaction processes that change the detected fluorescence of the sample. b) To obtain strong thermal coupling, the sample solution inside a capillary was placed between a temperature‐controlled silicon wafer and a glass cover slip, immersed in oil and locally heated with an IR laser. Through the same objective, the fluorescence emission was detected by a photodiode.

**Figure 2:**
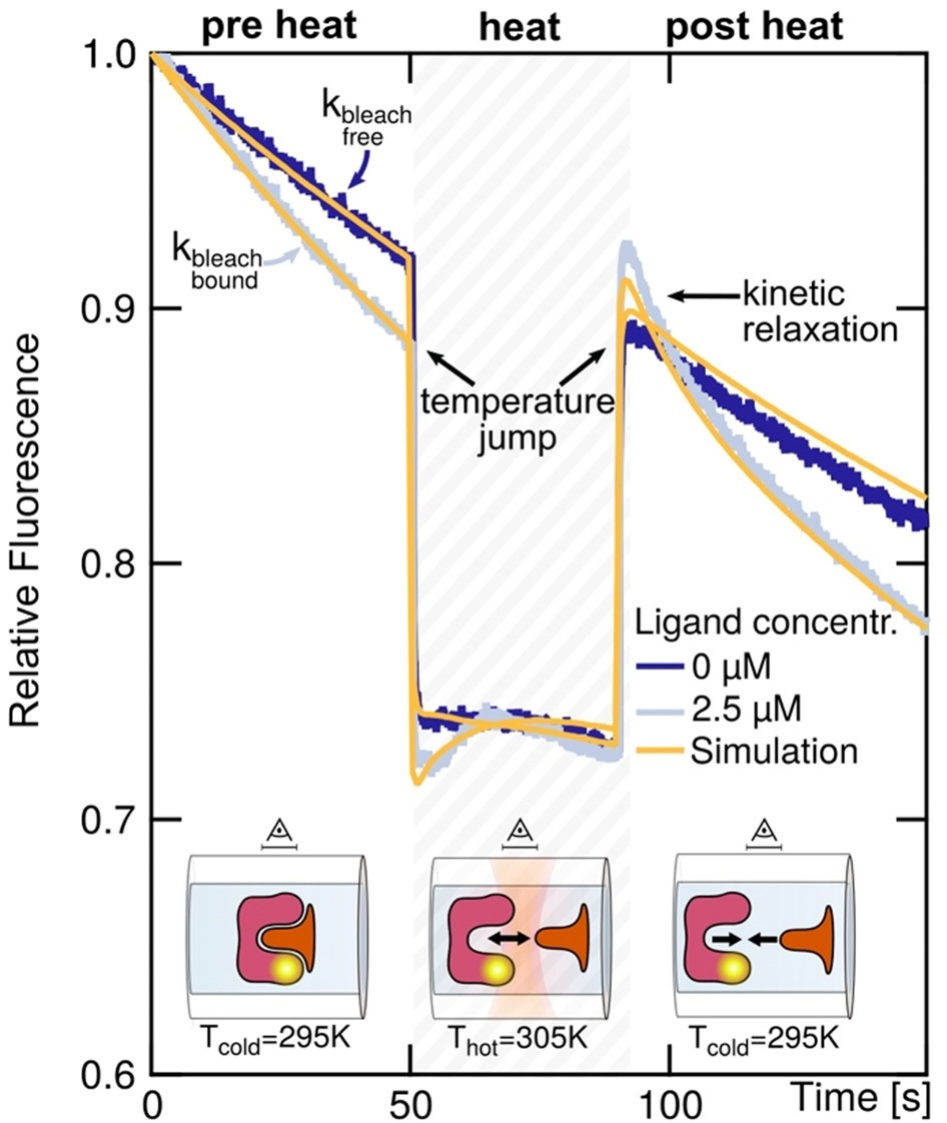
Fluorescence intensity unravels kinetics. In the pre heat phase, the fluorophore bleached due to LED illumination. The bleaching rate was higher for the bound complex (light blue). When the IR laser was switched on, the fluorescence quickly changed upon the temperature jump within 250 ms. In the subsequent heat phase, fluorescence was governed by unbinding kinetics, bleaching, convection and thermophoresis. When the laser was switched off, the sample quickly returned to ambient temperature. In this so‐called post heat phase, fluorescence was governed by bleaching, diffusion and kinetic relaxation from the unbound state towards the bound state. Fluorescence intensities are shown for 0 μm and 2.5 μm of 12mer DNA strands (dark and light blue) at 19 °C with 2 nm complementary labeled binder strand and COMSOL simulations (yellow), respectively.

**Figure 3:**
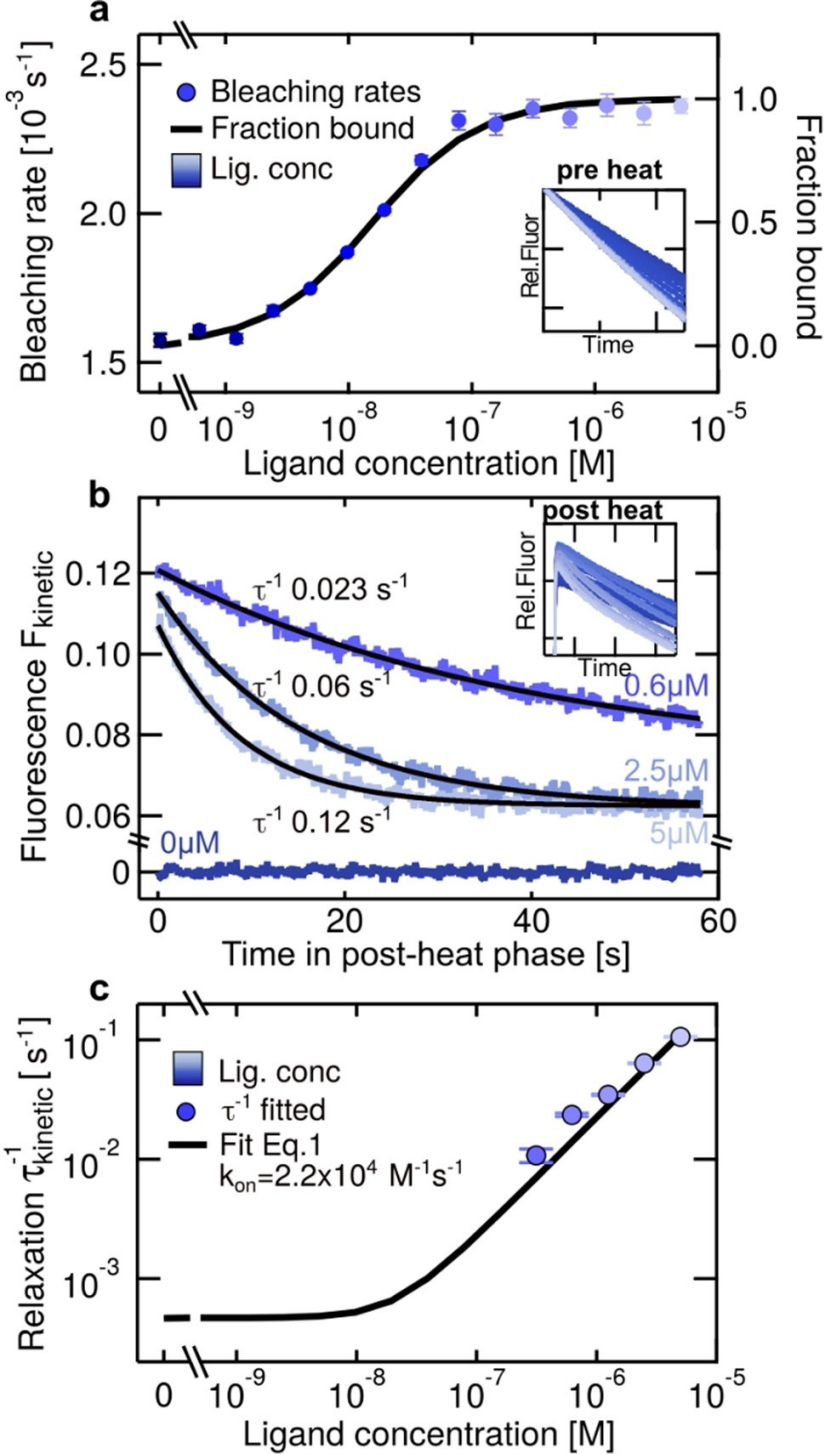
Kinetic data extraction. a) The binding curve and *K*_d_ were derived by plotting the bleaching rate in the pre heat phase against the total ligand concentration *L*_tot_ and fitting according to Equation (1(SI)). b) Kinetic relaxation was extracted by analyzing the bleach‐ and diffusion‐corrected fluorescence intensities of different *L*_tot_ in the post heat phase. The insets show all measured fluorescence signals of one dilution series. c) The fitted $\tau_{kinetic}^{-1}$ were plotted over *L*_tot_ to fit the on‐rate according to Equation (2(SI)). The data is shown for a fully complementary 12mer in 0.1×PBS at 16 °C, resulting in *k*_on_=2.2×10^4^ m ^−1^ s^−1^ and *k*_off_=2.4×10^−4^ s^−1^.

**Figure 4:**
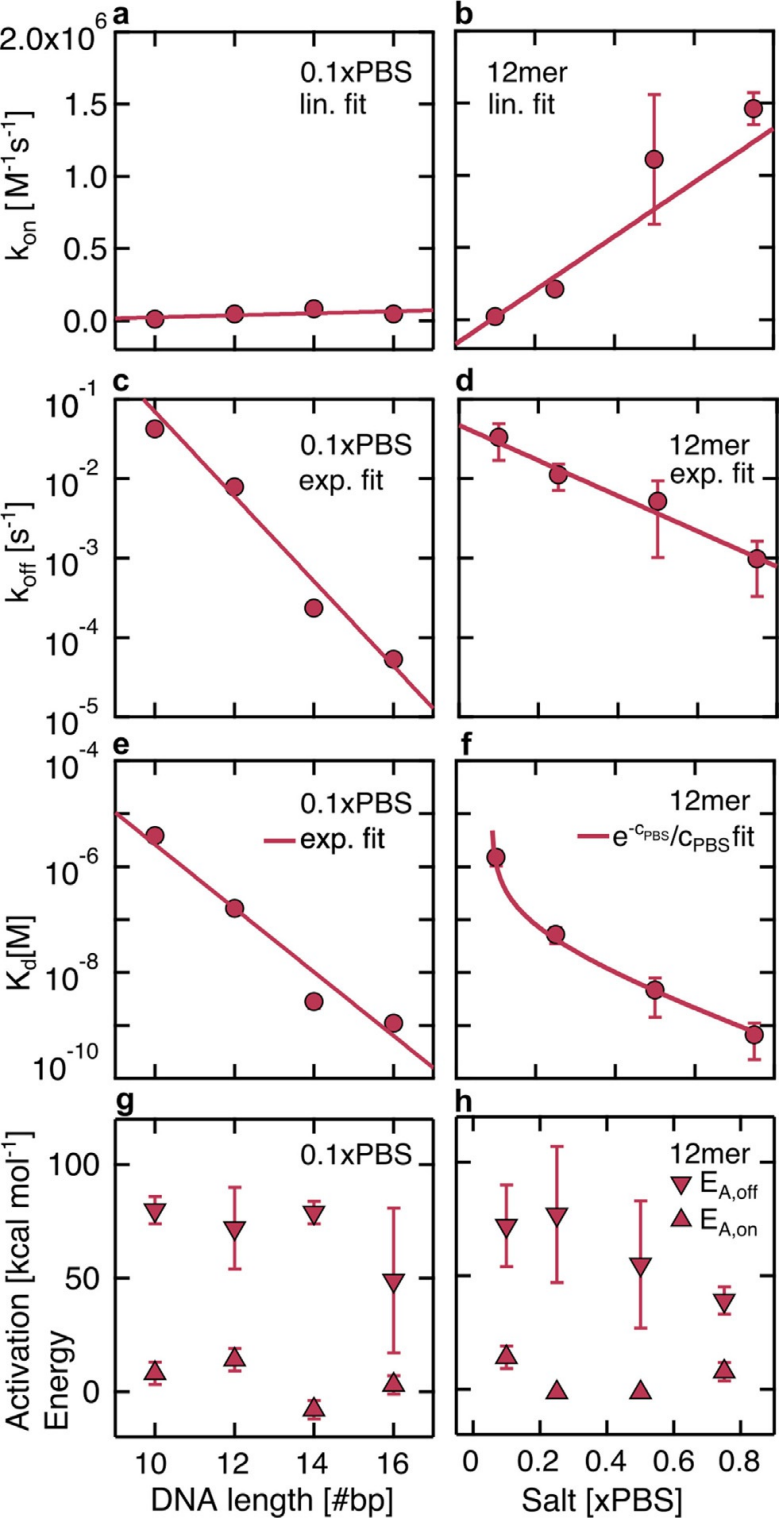
Dependence of *k*_on_, *k*_off_, *K*_d_ and *E*_A_ of complementary DNA on strand length and salt concentration. a) The on‐rate did not show strand length dependence but b) linear salt dependence. The off‐rate decreased exponentially with c) strand length and d) salt concentration. e) The resulting dissociation constant *K*_d_=*k*_off_/*k*_on_ decreased exponentially with length and f) according to $K_{d}\propto e^{-c_{\mathrm{PBS}}}/c_{\mathrm{PBS}}$ with PBS concentration. g,h) Arrhenius activation energy *E*_A_ for on‐rate and off‐rate. Length (salt) dependence was measured at 22 °C (25 °C).

**Figure 5:**
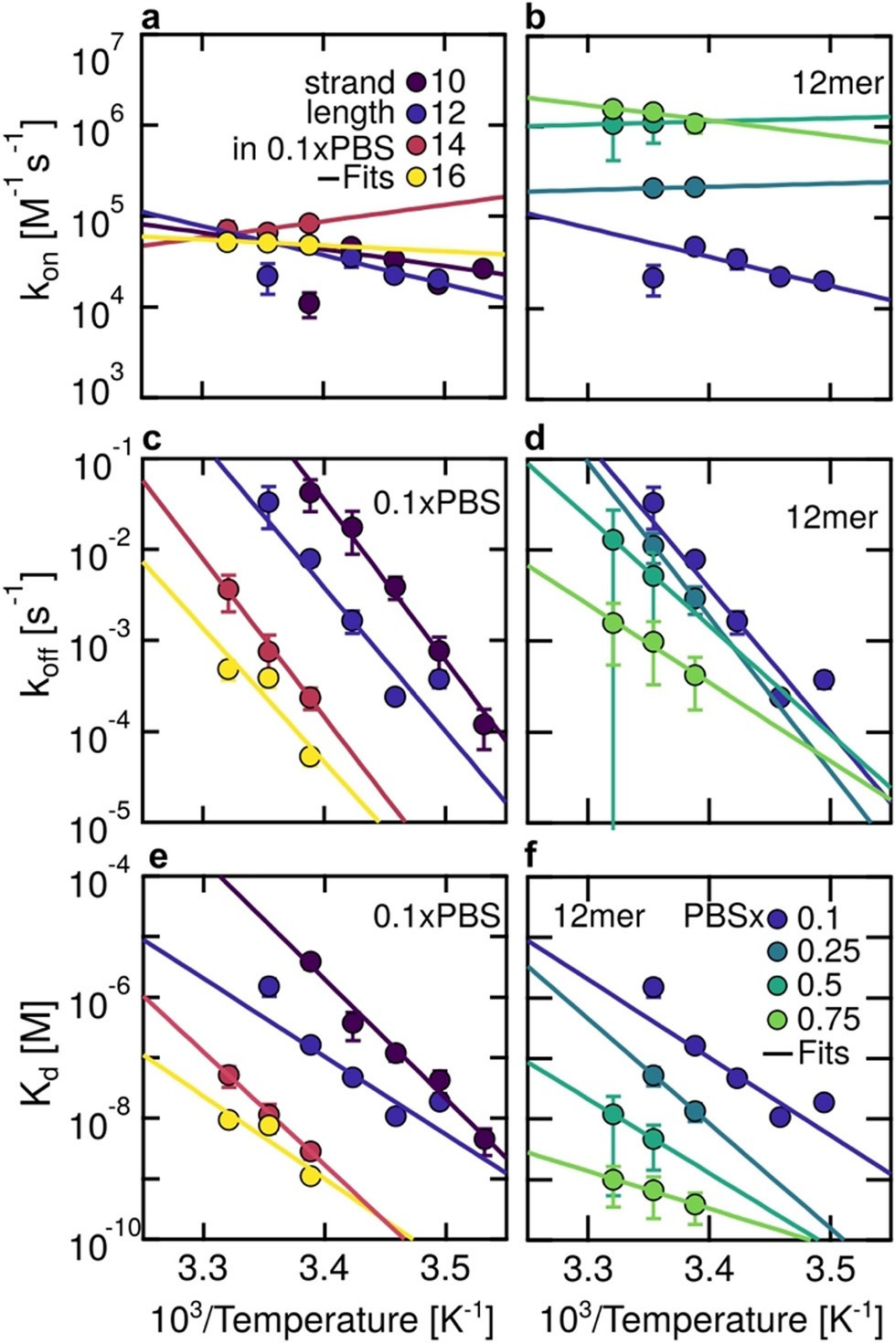
Temperature dependence of *K*_d_, *k*_off_ and *k*_on_ of fully complementary DNA strands. a–d) Eyring plots of transition state theory of on‐rates and off‐rates. a,b) On‐rates showed no strong dependence on temperature. c,d) The corresponding off‐rates showed an exponential decrease with 1/*T*. e,f) The Van't Hoff plots showed the expected exponential decrease of *K*_d_ with 1/*T*.

**Figure 6:**
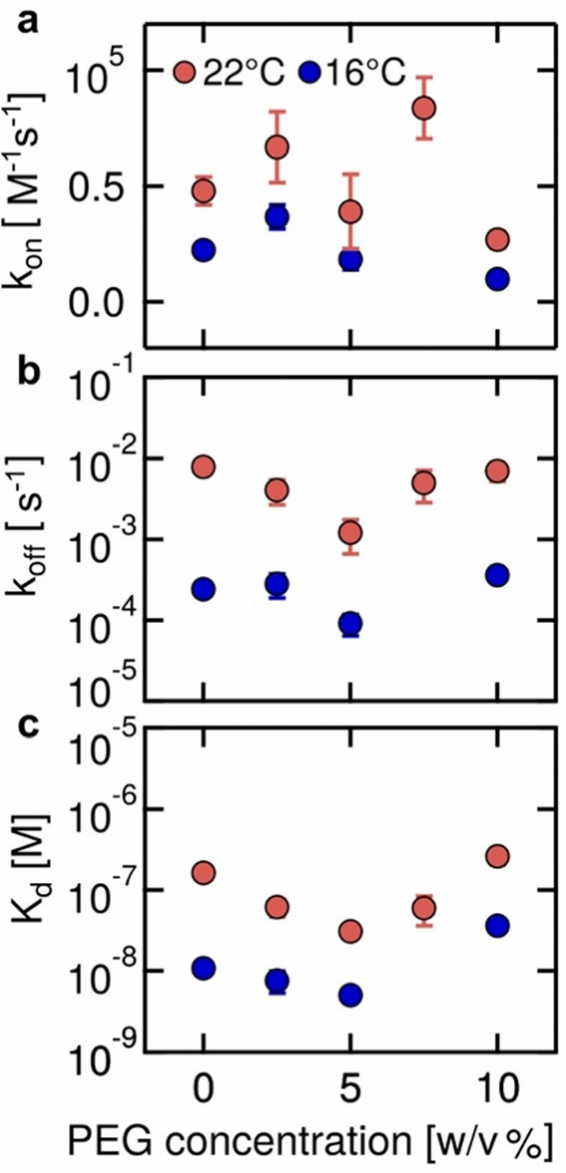
Hybridization rates *k*_on_, *k*_off_ and *K*_d_ of fully complementary 12mer DNA strands in crowded solutions with PEG 8000. a) The on‐rates, b) the off‐rates and c) the resulting *K*_d_ showed weak dependence on PEG concentrations. All measurements were conducted in 0.1×PBS with 0.05 % Tween.

Here, we present a novel method called “kinetic microscale thermophoresis” (KMST) to experimentally determine kinetic reaction rates in bulk solution, in an all‐optical way that only requires minimal preparation steps. KMST is an extension of the well‐established and widely used microscale thermophoresis (MST) method.[^1^, ^21^, ^22^, ^23^, ^24^, ^25^, ^26^, ^27^] MST uses binding‐dependent intensity change of fluorescently labeled molecules in a microscopic temperature gradient to measure the binding affinity. MST can also detect minute changes in conformation, charge, and size upon binding or enzymatic activity.^[1]^ MST has been successfully used in the past to determine affinities in complex solutions.^[21]^ The KMST technique offers measurement of binding kinetics together with binding affinity in a single experimental run. This is achieved by increasing the thermal dissipation of the samples (Figure 1), making the MST setup capable of temperature jumps. Analysis of the temperature‐dependent features, including the bleaching, diffusion, thermophoretic and kinetic contribution to the fluorescence intensity (Figure 1 and Figure 2), allows for the determination of not only the binding affinity but also the kinetic rates in a single experiment (Figure 3). We measured the relaxation constants in the range of 0.01–0.5 s^−1^, allowing measurements of *k*_on_ values between 10^4^ and 10^6^ m ^−1^ s^−1^ and *k*_off_ between 10^−4^ and 0.1 s^−1^. Although these ranges do not cover the entire spectra of biomolecular on‐rates (10^3^–10^9^ m ^−1^ s^−1^) and off‐rates (10^−5^–1 s^−1^),^[28]^ the method provides a large enough interval to study many biomolecular systems. To demonstrate the effectiveness of the method, we systematically measured the kinetic hybridization rates of fully complementary DNA strands between 10 bp and 16 bp in varying salt concentrations (Figures 4 and 5). The off‐rates showed exponential dependence on strand length, temperature and salt concentration. The on‐rates showed weak dependence on strand length and temperature and linear dependence on salt concentration. Moreover, an analysis of the temperature dependence of the kinetic rates shed light on the hybridization mechanism of DNA and summarized the determinants of DNA binding. Our results on DNA hybridization show that KMST is a promising method to measure reaction kinetics without immobilization, with fluorescent labeling of only one binding partner and in crowded solutions (Figure 6).

## Results and Discussion

### Binding Kinetics from Kinetic Microscale Thermophoresis

We extended the conventional MST setup (*Nanotemper Monolith NT.115* ^*Pico*^) to kinetic MST by placing the sample‐containing capillary on a silicon wafer and immersing it in oil (Figure 1). The fluorescence excitation/detection unit of the NT.115^Pico^ measured the fluorescence intensity change over time at a certain spot of the sample (Figure 1 b). An infrared (IR) laser with an emission wavelength of 1480 nm was focused on the center of the capillary to create a temperature gradient within the capillary for a defined time period. The strong thermal coupling provided quick formation and reduction of the temperature gradient in less than 250 ms (SI‐Figure 1). Averaged over the volume, the temperature gradient spanned about 10 K and led to convection and thermophoretic movement of the binder and the ligand^[1]^ (SI‐1).

The binding affinity *K*_d_ and the kinetic parameters *k*_on_ and *k*_off_ were obtained from the fluorescence intensity measurements of a dilution series with a constant amount of (labeled) binder B*tot =2 nm and increasing concentration of the ligand *L*_tot_. Each measurement could be divided in three successive phases (Figure 2). In the pre heat phase, the reaction system was at (thermal) equilibrium and the fluorescence intensity was only governed by the binding‐dependent photobleaching rates *k*_bleach_ of the sample. The *K*_d_ and the binding curve were determined by plotting *k*_bleach_ over *L*_tot_ and fitting the data to Equation (1(SI))^[24]^ (Figure 3 a and SI‐2). In the successive heat phase, the sample was heated by the IR laser for 40 seconds, leading to dissociation of the complex and rapid decrease in fluorescence due to the temperature dependence of the dye.^[26]^ In this phase, the fluorescence intensity of the system was governed by thermophoretic movement, convection, bleaching and kinetics, thus not reliable enough to derive the kinetic fingerprint (SI‐3). When the IR laser was switched off, the system returned to thermal equilibrium within 250 ms. Subsequently, dissociated binder and ligand re‐associated and the kinetic fingerprint could be derived in this so‐called post heat phase by dissecting kinetics from the bleaching and diffusion contributions to the fluorescence. The bleaching and diffusion contributions were elucidated from pre heat phase and the zero‐ligand sample ($L_{\mathrm{tot}}=$ 0 m and $B_{\mathrm{tot}}{}^{*}=$ 2 nm) in the post heat phase, respectively. Then, the fluorescence intensities of the post heat phase were corrected for bleaching and diffusion for each ligand concentration and exponential kinetic relaxation $\propto \exp\left(-t/\tau_{\mathrm{kinetic}}\right)$ was fitted using Equation (7(SI)) (Figure 3 b and SI‐4). The resulting inverse kinetic relaxation constants $\tau_{\mathrm{kinetic}}^{-1}$ were plotted against the total ligand concentration and fitted according to Equation (2(SI)) to derive *k*_on_ (Figure 3 c), which was then used to calculate *k*_off_=*K*_d_ *k*_on_.

To confirm the experimental results, we performed finite element simulations using COMSOL Multiphysics, which captured the relevant interactions between heating, laminar flow, bleaching and reaction kinetics of diluted species in the sample capillary (yellow lines in Figure 2 and SI‐5). The simulated fluorescence intensities and the corresponding kinetic rates were similar to the ones determined by experiments. This suggests coherence of experimental observations and theoretical expectations based on fundamental rate equations.

KMST benefits from the advantages of the widely used MST technique:[^1^, ^21^, ^22^, ^23^, ^24^, ^26^] reliable and reproducible data acquisition, low cost and low sample consumption. Importantly, both methods rely on labeling of only one of the reactants (instead of both) which is less expensive, facilitates sample preparation and ultimately minimizes label‐related interferences in the binding process. KMST additionally offers determination of the kinetic rates along with the binding affinity in a single dilution series. A volume of less than 5 μL and around nm concentrations of labeled binder and down to μm concentrations of ligand substantially decrease the cost of the measurement.^[1]^ The additional features of KMST: the dilution series, the capillary filling, the placement of the capillaries on the silicon plate and immersion in oil do not require high‐precision handling. The subsequent data analysis is based on fundamental rate equations rather than complex theoretical models and is robust against uncertainties of individual capillaries. Moreover, due to its ease of use and fast preparation, KMST can also be used for high‐throughput *K*_d_ and kinetic rates determination.

The kinetic fingerprint deduction from KMST relies on a conformational change upon binding in the ligand–binder system. This leads to different absolute fluorescence levels (bound vs. unbound state) which were sufficient to detect the kinetic rates. In the probed system, the Cy5‐label was attached at the 5′‐end of a single strand DNA 16mer (binder). Complementary ssDNA strands of different lengths were used as the ligand. Our control measurements with a modified location of the fluorescent label that was more distant to the binding area resulted in similar affinities and kinetic rates (SI‐6). We conclude that the change of the electronic configuration of the fluorophore due to a distant binding was sufficient to detect binding, thus kinetics. We used simulation data to test the applicability of the method to systems with significant size difference between the reactants (SI‐7). The results suggest that the analysis is robust to reactants with significantly different sizes and the effects can be corrected by numeric simulations. The effects are minimized if the larger reactant is labeled.

We discuss four conditions which contribute to optimal experimental rate determination (SI‐8). First, for reliable fluorescence detection $B_{\mathrm{tot}}{}^{*}>$ 1 nm is optimal, allowing robust analysis of binding affinities $K_{d}>$ 1 nm. Second, the kinetic and temperature jump‐related components of the fluorescence had to be clearly separable in time, allowing for the study of systems with $\tau_{\mathrm{kinetic}}>$ 1 s. Third, since the measurements rely on temperature‐dependent (un)binding, the binder–ligand system needs to have a significant enthalpic contribution. Fourth, similar to every technique that relies on fluorescence imaging,[^5^, ^29^, ^30^] the quantum yield of the fluorescence label has to depend on binding for deriving the kinetic fingerprint from the fluorescence intensity.

The range of measurable on‐rates and off‐rates with KMST was comparable with that of label‐free methods, for example, the measurable ranges by SPR[^31^, ^32^] are 10^3^ m ^−1^ s^−1^–10^8^ m ^−1^ s^−1^ for *k*_on_ and 10^−6^ s^−1^–1 s^−1^ for *k*_off_. However, the limitations for measuring high on‐rates with KMST and SPR differ: while SPR is limited by mass transport^[33]^ and requires molecules with large molecular mass, KMST is limited by the speed of the temperature jump and small $K_{d}<$ 1 nm in combination with fast kinetics.^[34]^ With KMST, kinetic rates can be measured over a wide range of salt concentrations and in crowded solutions without significant loss of accuracy (see below). In contrast, with decreasing ionic strength the non‐specific electrostatic binding increases and changes the sensor response in surface‐based kinetic measurement methods.^[35]^ The kinetic rates for DNA hybridization vary significantly (up to several orders of magnitude) between different studies in the literature including ours.[^7^, ^9^, ^34^, ^36^, ^37^, ^38^, ^39^] This most probably originates from the fact that the kinetic rates strongly depend on the system parameters, for example, buffer, immobilization, fluorophore, temperature and other boundary conditions which vary remarkably among different studies.

### DNA Hybridization Kinetics

We measured hybridization kinetics of complementary DNA strands of different lengths with KMST under varying ionic strength and temperature conditions (SI‐9 and SI‐10). We also tried to get kinetic measurements of the same samples by using Eva Green intercalation dye in temperature jump experiments with a thermocycler (SI‐10). Although such measurements were successful for kinetic FRET measurements,^[40]^ the intercalating dye was unfortunately not suitable for kinetics measurements of the short DNA strands in our hands. The KMST‐measured on‐rates showed weak to no dependence on strand length and increased linearly with salt concentration by $\left(1.9\pm 0.2\right)\times \frac{10^{6}M^{-1}s^{-1}}{\times \mathrm{PBS}}$ (Figure 4 a,b). On the other hand, the measured off‐rates showed exponential dependence on strand length (characteristic length 0.81 bp) and salt concentration (characteristic concentration 0.19xPBS, Figure 4 c,d). This correlation was reflected in the relationship of the dissociation constant *K*_d_, with strand length (characteristic length 0.72 bp) and salt concentration $K_{d}\propto e^{-c_{\mathrm{PBS}}}/c_{PBS}$ (Figure 4 e,f). Direct comparison of the absolute values of the measured rates with other studies is difficult due to varying parameters between the systems. Instead, we compare the measured values in terms of order of magnitude, their dependence on the salt concentration, strand length and temperature.

Our results suggest DNA hybridization on‐rates at low salt concentrations to be in the range of 10^4^–10^5^ m ^−1^ s^−1^. The on‐rates linearly increase with salt concentration up to 10^6^ m ^−1^ s^−1^ for 0.75×PBS (Figure 4 b), as reported earlier.^[41]^ At high salt concentrations (1×PBS), SPR experiments measured on‐rates of 10^4^ m ^−1^ s^−1^,^[36]^ an order of magnitude smaller than our measurement. FRET measurements for 9mers reported on‐rates in the low range of 10^6^ m ^−1^ s^−1^ (in 50 mm HEPES),^[37]^ similar to our findings. Measurements with TOOL reported on‐rates in the order of 10^6^–10^7^ m ^−1^ s^−1^ for 12mer and 16mer complementary DNA strands,^[38]^ an order of magnitude larger than our results. At low salt concentrations (<0.1×PBS), FRET measurements reported for 10mers on‐rates of 10^4^ m ^−1^ s^−1^ (in 3 mm PB buffer),^[19]^ which were also reported with quartz crystal microbalance of immobilized 10mers (in 10 mm TRIS, 0.1 m NaCl),^[39]^ and are similar to our results. Multi‐channel graphene biosensors reported on‐rates of 10^5^ m ^−1^ s^−1^ for immobilized target strands,^[9]^ which is an order of magnitude higher than our findings.

We observed on‐rates to be independent of the strand length (Figure 4 a), as previously reported.^[7]^ However, literature also reports the opposite:[^19^, ^38^, ^39^] Bielec et al. argue that the higher total charge of the longer strands pose a higher energetic barrier for hybridization, especially for low ionic salt environments.^[19]^ We tested a strand length difference of 6 up to a total length of 16 bases; these values may be too low to observe strand‐dependent on‐rates. Because Okahata et al.^[39]^ used immobilized probes, direct comparison is unfortunately limited.

Literature reported both smaller and larger off‐rates of DNA hybridization at low and high ionic strengths than our results. At low salt concentrations (<0.1×PBS), FRET measurements of Bielec et al.^[19]^ reported off‐rates two orders of magnitude smaller than ours. Morrison et al.^[7]^ found higher off‐rates at much higher salt concentrations of 10×PBS in temperature jump experiments with FRET pairs. Tawa et al.^[36]^ measured smaller off‐rates for longer strands at higher salt concentrations. Our measured off‐rates showed an exponential decrease with salt concentration (Figure 4 d), which was also reported by Okahata et al.^[39]^ and qualitatively supported by Braunlin et al.^[41]^ Similarly, the exponential decrease of the off‐rates with strand length (Figure 4 c) is in agreement with other studies.[^7^, ^34^, ^39^, ^42^]

### DNA Hybridization Thermodynamics

The measurements of the binding affinity and the kinetic rates at various temperatures allowed us to perform a thermodynamic analysis. The Van't Hoff plot was calculated by Equation 1 using the standard enthalpy Δ*H* ^0^ and standard entropy Δ*S* ^0^, which were fitted to *K*_d_ values of Figure 5 e,f under $K_{d}^{0}=$ 1 m standard conditions at 295 K, see Table 1 (*R*=1.987 cal K^−1^ mol^−1^ is the gas constant). *T*Δ*S* ^0^ and the Gibbs free energy Δ*G* ^0^=Δ*H* ^0^−*T*Δ*S* ^0^ were calculated. Increasing temperature destabilized the bound state and increased *K*_d_. The negative slope and positive intercept of the Van't Hoff fits yielded for Δ*H* ^0^<0 and Δ*S* ^0^<0.

(1) $$\ln\left(K_{d}^{0}/K_{d}\right)=\frac{-\Delta H^{0}}{RT}+\frac{\Delta S^{0}}{R}$$

**Table 1:** Van't Hoff parameters from Figure 5 e,f. Δ*H* ^0^, Δ*S* ^0^ were fitted to $ln\left(K_{d}^{0}/K_{d}\right)=\frac{-\Delta H^{0}}{RT}+\frac{\Delta S^{0}}{R}$ .

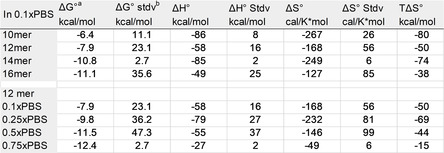

[a] Δ*G* ^0^ and *T*Δ*S* ^0^ were calculated. [b] The error was calculated by Gaussian error propagation. All values refer to standard temperature 298 K.

The Van't Hoff plots suggest Δ*H* ^0^ to be in the range of about −60 to −80 kcal mol^−1^ and Δ*S* ^0^ between −170 and −270 cal K^−1^ mol^−1^, which were also reported by surface‐tethered FRET measurements^[37]^ and are slightly above the values reported for 8mers by NMR.^[41]^ Additional melting curve measurements of the 12mer strands and associated Van't Hoff analysis showed similar *K*_d_ dependence on inverse temperature and similar Δ*H* ^0^ (SI‐11). At room temperature, enthalpic and entropic contributions nearly cancel each other resulting in small negative Δ*G* ^0^, supporting that DNA hybridization is a spontaneous process:[^37^, ^43^] the hydrogen bond formation and base stacking lead to release of heat and decrease in entropy due to reduced conformational flexibility in the bound state.[^44^, ^45^] Increased ionic strength increased both Δ*H* ^0^ and Δ*S* ^0^. However, Δ*H* ^0^ increased more than *T*Δ*S* ^0^, resulting in a higher net negative Δ*G* ^0^, thus favoring binding. The significance of Δ*G* ^0^ is limited due to large propagating uncertainties, see SI‐11. With increasing strand length, the increase in Δ*H* ^0^ and Δ*S* ^0^ resulted in a decrease of Δ*G* ^0^, thus favoring the hybridized state, similarly reported before.^[37]^

The measured temperature dependence of the on‐rates and off‐rates allowed us to determine the Arrhenius activation energies *E*_A,on_ and *E*_A,off_ (Figure 4 g,h) through $k=A\exp\left(-E_{A}/RT\right)$ , where *A* is the pre‐exponential factor and *k* is either the on‐ or off‐rate (SI‐12). The Arrhenius plots are shown in Figure 5 a–d. The on‐rates showed no or slight increase with temperature (Figure 5 a,b), resulting in small positive *E*_A,on_. The temperature dependence of on‐rates of DNA hybridization is still a matter of open debate. For *T*<*T*_melt_, literature reports increasing,^[7]^ decreasing^[34]^ and also non‐monotonic[^39^, ^46^] behavior. Our findings suggest that *E*_A_ slightly above or below zero cannot be used to rule out either of the hypotheses. *E*_A,on_ showed no significant dependence on strand length or salt concentration (Figure 4 g,h). The off‐rates showed the expected exponential dependence on inverse temperature[^7^, ^34^, ^39^, ^42^] (Figure 5 c,d). The measured *E*_A,off_ became smaller with increasing strand length and salt concentration (Figure 4 g,h). This is consistent with the view that the electrostatic repulsion between the negative chains of the DNA strands decreases at high salt concentrations, stabilizing the hybridized state.^[39]^ Similar behavior was observed for DNA hairpins.^[47]^

The identification of the Arrhenius activation energies with the thermodynamic quantities of the Eyring–Polanyi equation (*E*_A,on_≡Δ*H* ^≠^_on_ and *E*_A,off_≡Δ*H* ^≠^_off_) allowed a connection of kinetic quantities with thermodynamic quantities.[^37^, ^43^] The thermodynamic enthalpy and entropy landscapes of free, transition, and bound state could be determined (SI‐12). However, due to conceptual difficulties,^[48]^ the interpretation of the resulting Δ*H* ^≠^ and Δ*S* ^≠^ is limited.

### DNA Hybridization Kinetics in Crowded Solutions

Like most physiological processes, DNA hybridization takes place in crowded environment. However, measurements in complex solutions are typically experimentally more challenging. To simulate crowded environment, we used polyethylene glycol (PEG) 8000, which was used in earlier studies to simulate molecular crowding.[^38^, ^49^] As shown in Figure 6, the on‐rate, off‐rate and *K*_d_ did not show a clear relationship between PEG concentration and hybridization rates (SI‐13). Our results agree with other measurements by FRET[^38^, ^50^] that showed weak or no dependence of the DNA hybridization time constants on crowding agent concentrations. These results indicate that KMST, like other methods,[^38^, ^49^, ^50^] is a versatile technique which is able to measure kinetic reaction rates and binding affinity at different ionic strengths and in crowded environments.

## Conclusion

Herein, we have shown that combining MST with the temperature jump technique provides a novel method to determine the kinetic rates along with binding affinities in a single experiment. A simple hardware modification of a conventional MST setup to increase the thermal dissipation of the sample is sufficient to deduce kinetic relaxation from the fluorescence intensities. We systematically investigated the dependence of kinetic parameters of DNA hybridization on strand length, temperature and ionic strength. We found an exponential dependence of the off‐rate on strand length, salt and inverse temperature. We also showed no or weak dependence of the on‐rate on temperature and strand length and a linear dependence on salt concentration. These results did not only show the power of the kinetic MST as a method but also shed light on the hybridization mechanism of DNA. Unlike several other methods, labeling of only one of the reactants is sufficient, reducing the cost and time required as well as the label‐related interferences to the binding. The setup is very easy to use, robust and provides reliable and reproducible results. While the binding reaction of interest must have a sufficient enthalpic contribution, no artifact‐inducing processes, such as immobilization, are required. We believe that KMST could be of great interest to a broad audience and could offer new opportunities in biological and medical sciences.

## Conflict of interest

The authors declare no conflict of interest.

## Supporting information

As a service to our authors and readers, this journal provides supporting information supplied by the authors. Such materials are peer reviewed and may be re‐organized for online delivery, but are not copy‐edited or typeset. Technical support issues arising from supporting information (other than missing files) should be addressed to the authors.

Supplementary
