# Supplementary material for: Kinetic Microscale Thermophoresis for Simultaneous Measurement of Binding Affinity and Kinetics
Source: Angew Chem Int Ed Engl. 2021 May 11;60(25):13988–95. doi: 10.1002/anie.202101261 (PMC8251828; doi:10.1002/anie.202101261)
Supplement: Supplementary file 1 — Supplementary [file ANIE-60-13988-s001.pdf]

Angewandte Chemie
Eine Zeitschrift der Gesellschaft Deutscher Chemiker

Supporting Information

Kinetic Microscale Thermophoresis for Simultaneous Measurement of Binding Affinity and Kinetics

*Julian A. C. Stein, Alan Ianeselli, and Dieter Braun**

anie_202101261_sm_miscellaneous_information.pdf

Table of Contents

1 Experimental setup
2 Binding kinetics from bleaching rates
3 Choice of post heat phase for kinetic analysis
4 Reaction kinetics from fluorescence intensities
5 Rate equation simulations in 3D inside the capillary
6 Independence of kinetic rates on label site
7 Influence of diffusion on fluorescence analysis
8 Optimal conditions for KMST measurements
9 DNA samples & preparation
10 Summarized measured kinetic rates and dissociation constants
11 Van 't Hoff analysis
12 Thermodynamic analysis
13 Kinetic rates in crowded solutions

Experimental Procedures

1 Experimental setup

All KMST measurements were carried out with a Nanotemper Monolith(R) NT.115 Pico. Each sample of the dilution series was filled in a high precision round borosilicate glass capillary ID300µm, OD400µm, (Hollow Round Glass Capillaries, CM Scientific). The fluorescence excitation/detection unit of the NT.115 Pico measured the fluorescence intensity change with the RED filter set (excitation 605-645 nm, emission 660-710 nm) over the whole experiment time in a localized spot with a spatial range of about 20 µm.

The heating of the sample to the final temperature showed an exponential time-characteristic, with the inverse heating time constant τ_{heat}^{-1}, see SI-Fig.1 a & b. For MST measurements with conventional sample holder the capillary was freely-lying in air. The inverse heating time was $\tau_{heat}^{-1} = 0.38\,s^{-1}$, which corresponds to at heating time of 2.6 seconds. This results from the rather weak thermal coupling of the capillaries with the surrounding air and turned out to be is too slow for KMST detection.

To enable the detection of kinetic fingerprints, the thermal coupling of a KMST setup was strongly increased in comparison to an MST setup: first, the samples were loaded into smaller round glass capillaries with an inner diameter of 300µm OD 400µm, instead of conventionally used 500µm ID, 1000µm OD. Second, the commercially available sample holder for the NT.115 Pico was slightly modified: Instead of being surrounded by air, the capillaries containing the sample solutions were placed on a thin silicon wafer. Third, the capillaries on the silicon wafer were immersed with oil (Zeiss Immersion Oil 518 F). Fourth, a thin glass cover slip (Carl Roth) is placed on top of all capillaries, see Fig.1 a & b and SI-Fig.1 d. The covering of the capillaries by immersion oil and the glass cover slip yielded for an equal heat transfer and for a homogeneous fluorescence detection for of the 16 capillaries within the assay, respectively (not shown). For this, a thin section had to be milled out from top of the aluminum sample holder. The high thermal conductivity of the modified KMST setup provided much faster heat transfer from the heated sample to the environment and the inverse heating time increases to $\tau_{heat}^{-1} \approx 4\,s^{-1}$, corresponding to <250 ms heating time. It is an order of magnitude faster in comparison to a standard sample holder, see SI-Fig.1 b. It is also an order of magnitude faster than the fastest measured kinetic relaxation time constants.

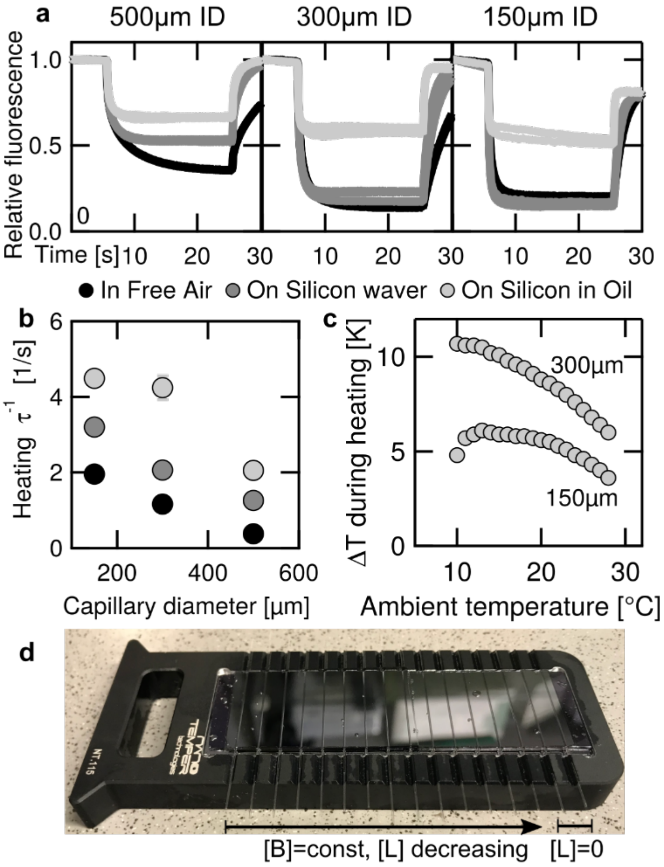

SI-Figure 1: ***Sample heating characteristics a*** *Relative fluorescence over time of Cy-5 in 1xPBS solution during heating for various capillary sizes (500µm, 300µm and 150µm inner diameter) and sample holder specifications.* ***b*** *The strong heat-coupling of the sample to the Silicon-Oil-Coverslip holder (SOC) enables for short high inverse heating times.* ***c*** *the temperature jump during the hot time is about 10K for capillaries with inner diameter 300µm.* ***d*** *The SOC sample holder with capillaries filled with constant binder and decreasing ligand concentrations - from left to right*

To determine the amplitude of the temperature jump during the heating, the fluorescence dependence of the dye was measured for temperatures between 10°C and 28°C. The absolute fluorescence of Cy5-only samples (biomers GmbH, Ulm, Germany, 10 nM in 0.1xPBS buffer, Ambion) during the hot time are related to the initial fluorescence at the measured ambient temperatures. The absolute

fluorescence for the hot time depended linearly on the ambient temperature for labeled DNA and Cy5 only in the measured range 10°C to 28°C (not shown). The amplitude of the temperature jump is shown in SI.Fig.1 c for various ambient temperatures. We found a temperature jump of about $\Delta T = 10\ K$ for 300µm ID round capillaries and $\Delta T = 6\ K$ for 150 µm capillaries for Cy5-only measurements. These temperature jumps were the average over the detection volume. Measurements of samples with the Cy5-labeled 12mer DNA strand showed a highly similar behavior (not shown). Due to restrictions of the adjustment of the ambient temperature, all obtained hot time values above 20°C were made on the assumption that the fluorescence changes from cold to hot decreased linearly with temperature.

The use of smaller capillaries led to less pronounced convection effects, which interfered with the measurement of kinetics in the following way: During heating, the movement of (bleached) fluorescent molecules out of the (warmer) illuminated region towards the (colder) non-illuminated region and the influx of non-bleached fluorescent molecules into the heated region change the fluorescence independently from kinetic processes. When the laser was switched off, fluorescent molecules from formerly not heated regions could diffuse back into the illuminated region and change the detected fluorescence. Small capillaries reduced these effects. The small capillary size also decreased the thermophoretic effect due to more shallow temperature gradients.

2 Binding curve from bleaching rates

The in equilibrium, the reaction $L + B^* \Leftrightarrow LB^*$ of a labeled binder B^* and ligand L can be characterized by $K_\mathrm{d} = \frac{L \cdot B^*}{LB^*} = \frac{k_\mathrm{off}}{k_\mathrm{on}}$ with L the free ligand and B^* the free binder concentrations and LB^* the bound complex concentration. The concentrations of free L and free B^* are in general not experimentally accessible, but only the total ligand concentration L_{tot} and total binder concentration B^*_{tot}. The on-rate k_{on} and off-rate k_{off} correspond to the respective equilibrium conditions. The fraction of bound complex $P_{bound}=LB^*/B^*_{tot}$ can be expressed by[1]

$$P_\mathrm{bound} = \frac{LB^*}{B^*_\mathrm{tot}} = \frac{L_\mathrm{tot}+B^*_\mathrm{tot}+K_\mathrm{d}-\sqrt{\left(L_\mathrm{tot}+B^*_\mathrm{tot}+K_\mathrm{d}\right)^2-4L_\mathrm{tot}\cdot B^*_\mathrm{tot}}}{2B^*_\mathrm{tot}} \tag{1}$$

The measured bleaching rates k_{bleach} are related to the fraction bound by

$$P_\mathrm{bound} = \frac{k_\mathrm{bleach} - k_\mathrm{bleach,bound}}{k_\mathrm{bleach,free} - k_\mathrm{bleach,bound}}$$

Thus, the fitting the measured bleaching rates to the total ligand concentrations yields for the fraction bound, K_d, $k_{bleach,\ free}$ and $k_{bleach,\ bound}$.[2] As documented in the works of Schoen[3] a square root-dependence of $\tau^{-1}_\mathrm{kinetic} = \sqrt{\left(k_\mathrm{off}(k_\mathrm{off} + 4k_\mathrm{on}B_\mathrm{tot})\right)}$ was found for a 1:1 mixture of $B_{tot}=L_{tot}$. In our case, the mixture ratio deviated from the 1:1 ratio, therefore the relationship had to be adjusted as shown in Eq. 2, pointing to a linear dependence of $\tau^{-1}_{kinetic}$on the ligand concentration L_{tot} with constant binder B_{tot}.

3 Choice of post heat phase for kinetic analysis

In principle, the kinetic rates could have been determined by the analysis of the fluorescence intensity after both temperature jumps. The analysis of the fluorescence during the post heat phase after the second temperature jump was suitable for two reasons. First, the determination of the kinetic rates with Eq. 2 required the concentrations of the reactants to reach equilibrium, that was at constant temperature of the sample in the post heat phase. The determination of K_d and the respective binding curve in the pre heat phase allowed for determination of this equilibrium state. Pre heat and post heat phases (after kinetic equilibration) have the same equilibrium. Second, the fluorescence change during the hot phase due to strong convection and thermophoretic effects superimposed with the fluorescence change due to the kinetic effects and bleaching. The disentanglement of the effects from the fluorescence intensity in this phase was difficult. As seen in Fig.2, our numerical model could handle all these effects in a reasonable manner, however these results were not sufficient to perform a full and reliable kinetic analysis.

4 Reaction kinetics from fluorescence intensities

The on-rate of a ligand-binder reaction $L + B \Leftrightarrow LB$ was fitted to the experimentally-accessible kinetic time constant

$$\tau^{-1}_\mathrm{kinetic} = k_\mathrm{off} + k_\mathrm{on}(L + B^*) = k_\mathrm{on}\sqrt{(L_\mathrm{tot} + B^*_\mathrm{tot} + K_\mathrm{d})^2 - 4L_\mathrm{tot} \cdot B^*_\mathrm{tot}} \tag{2}$$

with the dissociation constant K_d and the known total binder B^*_{tot} and total ligand L_{tot} concentrations for a second-order reaction process. The free L and free B^* concentrations were expressed by the fraction bound from Eq.1 and the relation $K_d=k_{off}/k_{on}$ was used. K_d and the fraction bound in equilibrium $P_{bound,eq}$, were determined by the binding-dependent fluorescence photobleaching in the pre heat phase,[4] see SI-2. We fitted the on-rate to a dilution series with increasing L_{tot}, constant label concentration $B^*_{tot}<K_d$ and the exponentially fitted $\tau^{-1}_{kinetic}$from the post heat phase. The usage of multiple L_{tot} increased the robustness of the fit due to more measurement points within one single concentration k_{on}-fit.

To access the kinetic relaxation constant $\tau^{-1}_{kinetic}$in the post heat phase, we dissected the kinetic contribution from bleaching and convection terms within the fluorescence intensity. We analyzed the fluorescence intensity $F(t)[\#emitted\ photons/second]$ per time interval Δt as the integral over the illuminated detection volume dV of the time and space-dependent free $B^*(t,\vec{x})[mol/m^3]$ and bound $LB^*(t,\vec{x})[mol/m^3]$ fluorescent molecules – B and LB denote the bleached species which did not contribute to the fluorescence signal:

$$F(t) = \int dV \left(B^*(t,\vec{x}) \cdot \left(F_\mathrm{Free} + \frac{\partial F_\mathrm{Free}}{\partial T} \cdot \Delta T\right) + LB^*(t,\vec{x}) \cdot \left(F_\mathrm{Bound} + \frac{\partial F_\mathrm{Bound}}{\partial T} \cdot \Delta T\right)\right) \tag{3}$$

With $F_i \left[\frac{\text{#emitted photons}}{second \cdot mol}\right]$ the quantum yields of bound and free binder states, $\frac{\partial F_i}{\partial T}$ the respective temperature dependence and $\Delta T = T - T_0$ the temperature change compared to the equilibrium temperature T_0. The concentrations of ligand, free and bound (bleached) binder depended on kinetics, diffusive/convective movement and bleaching, see Fig.1 a. For any point $\vec{x}$, the following rate equations applied

$$\begin{aligned}
\dot{L}(t) &= +k_{\text{off}} \cdot LB^*(t) - k_{\text{on}} \cdot B^*(t) \cdot L(t) + D\Delta L(t) - \nabla\left(L(t) \cdot \left(\vec{u} - D_{T,L} \cdot \nabla T\right)\right) \\
\dot{B^*}(t) &= +k_{\text{off}} \cdot LB^*(t) - k_{\text{on}} \cdot B^*(t) \cdot L(t) + D\Delta B^*(t) - \nabla\left(B^*(t) \cdot \left(\vec{u} - D_{T,B} \cdot \nabla T\right)\right) - k_{\text{bleach,free}} \cdot B^*(t) \\
\dot{LB^*}(t) &= -k_{\text{off}} \cdot LB^*(t) + k_{\text{on}} \cdot B^*(t) \cdot L(t) + D\Delta LB^*(t) - \nabla\left(LB^*(t) \cdot \left(\vec{u} - D_{T,LB} \cdot \nabla T\right)\right) - k_{\text{bleach,bound}} \cdot LB^*(t) \\
\dot{B}(t) &= +k_{\text{off}} \cdot LB(t) - k_{\text{on}} \cdot B(t) \cdot L(t) + D\Delta B(t) - \nabla\left(B(t) \cdot \left(\vec{u} - D_{T,B} \cdot \nabla T\right)\right) + k_{\text{bleach,free}} \cdot B^*(t) \\
\dot{LB}(t) &= -k_{\text{off}} \cdot LB(t) + k_{\text{on}} \cdot B(t) \cdot L(t) + D\Delta LB(t) - \nabla\left(LB(t) \cdot \left(\vec{u} - D_{T,LB} \cdot \nabla T\right)\right) + k_{\text{bleach,bound}} \cdot LB^*(t)
\end{aligned} \quad (4)$$

With $k_{\text{bleach,i}}$ the bleaching rates of *i* the free and bound state, $D\Delta c - \nabla\left(c(t) \cdot \left(\vec{u} - D_T \cdot \nabla T\right)\right)$ the diffusive, advective and thermophoretic contributions with the diffusion constant D, the thermal diffusion constant D_T and the velocity field $\vec{u}$ for the respective concentration c. We rewrote the motion term as $D(t)$, which we approximate to be the same for B^* and LB^*. To extract the kinetic relaxation constant from the fluorescence intensities in the post heating phase, we wrote the solution B^* and LB^* of the equation system (Eq. 4) as a product of the kinetic, bleaching and convective solutions $B^*(t) = B^*_{tot} \cdot B_{\text{kinetic}}(t) \cdot B_{\text{bleach}}(t) \cdot B_{\text{diffusion}}(t)$. B^* and LB^* are expressed by the total concentration of labeled binder B^*_{tot} with $B^* = B^*_{tot} - LB^*$ and the fraction bound $LB^* = P_{\text{bound}}(t) \cdot B^*_{\text{tot}}$. The kinetic solution after a quick temperature jump is a second order exponential relaxation

$$B_{\text{kinetic}}(t) = 1 - P_{\text{bound}}(t) = 1 - \left(P_{\text{bound,eq}} - \left(P_{\text{bound,eq}} - P_{\text{bound,hot}}\right) \cdot \exp(-t/\tau_{\text{kinetic}})\right) \quad (5)$$

with the fraction bound in equilibrium$P_{bound,eq}$, the fraction bound in the hot phase $P_{bound,hot}$ and the kinetic relaxation constant from Eq. 2. The bleaching term was extracted from the pre heating phase and read $B_{\text{bleach}}(t) = \exp\left(-t \cdot k_{\text{Bleach,free}}\right)$ and $LB_{\text{bleach}}(t) = \exp\left(-t \cdot k_{\text{Bleach,bound}}\right)$. The diffusion term $B_{\text{diffusion}}(t)$ was obtained by the zero-ligand sample, see below. With $\hat{F}_i = \left(F_i + \frac{\partial F_i}{\partial T} \cdot \Delta T\right) \cdot B^*_{\text{tot}}$ the fluorescence in the post heat phase reads:

$$\begin{aligned}
F(t) &= \int dV\, D(t) \cdot (\hat{F}_{\text{Bound}} \left(P_{\text{bound,eq}} - \left(P_{\text{bound,eq}} - P_{\text{bound,hot}}\right) \cdot \exp(-t/\tau_{\text{kinetic}})\right) \cdot \exp\left(-t \cdot k_{\text{Bleach,bound}}\right) \\
&+ \hat{F}_{\text{Free}} \left(1 - \left(P_{\text{bound,eq}} - \left(P_{\text{bound,eq}} - P_{\text{bound,hot}}\right) \cdot \exp(-t/\tau_{\text{kinetic}})\right)\right) \cdot \exp\left(-t \cdot k_{\text{Bleach,free}}\right))
\end{aligned} \quad (6)$$

To access the time-dependent kinetic relaxation, the bleaching and diffusion contributions needed to be eliminated. The measured data was normalized to its initial value, and then Eq. 6 was divided by the exponential bleaching and diffusion contributions to obtain the kinetic term (Eq. 7). The exponential relaxation was fitted to Eq.7. Therefore, the effective bleaching rate k_{bleach} as an approximate for the underlying free and bound bleaching rates was obtained from equilibrium by fitting $F_{eq} \approx F_0 exp(-t \cdot k_{bleach})$ to the pre heat phase, see SI-2. The fit for this exponential bleaching did not have an offset, as bleaching converged to 0 for $t \to \infty$.

To clearly separate artifacts from the temperature jump to the fluorescence intensity from kinetics in time,, the first 2.1 seconds after the detection of the second temperature jump were cut out. The diffusion term D(t) was obtained from the zero-ligand fluorescence in the post heat phase $F_{\text{Ligand=0}}(t) = \int dV \cdot D(t) \cdot \hat{F}_{\text{Free}} \cdot \exp\left(-t \cdot k_{\text{Bleach,free}}\right)$. Yet, we could only determine $D(t) \cdot \hat{F}_{\text{Free}} \approx F_{\text{ligand=0}} / \exp\left(-t \cdot k_{\text{Bleach,free}}\right)$ which was sufficient for further analysis. Dividing F(t) in the post heat phase by the effective bleaching contribution $\exp\left(-t \cdot k_{\text{Bleach,effective}}\right)$ and $D(t) \cdot \hat{F}_{Free}$ yielded for the kinetic fluorescence term

$$F_{\text{kinetic}}(t) = \int dV \left(1 - P_{\text{bound,eq}} + \frac{\hat{F}_{\text{Bound}}}{\hat{F}_{\text{Free}}} P_{\text{bound,eq}} + \frac{\hat{F}_{\text{Free}} - \hat{F}_{\text{Bound}}}{\hat{F}_{\text{Free}}} \left(P_{\text{bound,eq}} - P_{\text{bound,hot}}\right) \cdot \exp(-t/\tau_{\text{kinetic}})\right) \quad (7)$$

The right hand side of the equation has only one time-dependent term: the kinetic relaxation term $\exp(-t/\tau_{\text{kinetic}})$. We subtracted 1 from the right hand side of Eq. 7, also see SI-Fig.2 a, and fitted an exponential function $F_{kinetic}(t) = F_{\text{kinetic,offset}} + F_{\text{kinetic,amplitude}} \cdot \exp(-t/\tau_{\text{kinetic}})$ to obtain $\tau^{-1}_{\text{kinetic}}$, see Fig.3 b. The subtraction of 1 yielded for better converging fits but is in general not necessary. We did not further investigate $F_{\text{kinetic, offset}}$ as it carried no information of interest of the kinetic relaxation. The errors of the $\tau^{-1}_{\text{kinetic}}$fit (which were later used for the weights of the on-rate fit) were obtained by the root mean squared error times the variance of the fit.

The on-rate was fitted with a Levenberg-Marquart algorithm according to Eq. 2 for the fitted$\tau^{-1}_{kinetic}$and respective total ligand concentrations L_{tot}. K_d and labeled binder concentrationB^*_{tot}were constant for all ligand concentrations and k_{on} was the only fitting parameter. The fitting-weights ω for each data point the inverse quadratic relative errors $\omega = \frac{1}{\Delta\tau^{-2}_{\text{kinetic}} / \Sigma \Delta\tau^{-2}_{\text{kinetic}}}$, with the fitting errors $\Delta\tau^{-1}_{\text{kinetic}}$. If the fluorescence intensity of a capillary (with L_{tot} concentration) did not show kinetic behavior, the fitted relative error $\frac{\Delta\tau^{-1}_{\text{kinetic}}}{\tau^{-1}_{\text{kinetic}}}$ was comparably large (>0.15). Those L_{tot} concentrations were excluded from the on-rate fit. The off-rate k_{off} was calculated by $k_{\text{off}} = K_{\text{d}} \cdot k_{\text{on}}$ and the error of the off-rate was obtained by Gaussian error propagation from K_d and k_{on} fits. All analysis calculations were carried out in LabView.

5 Rate equation simulations in 3D inside the capillary

To validate the experimentally-determined kinetic rates, we simulated the reaction kinetics with finite elements simulations (COMSOL Multiphysics), also see separate .mph file. We simulated the fluorescence intensity of the ligand-binder system with the measured kinetic rates and bleaching rates on the basis of the fluorescence model. So we could check if the analysis of the kinetic rates that resulted from the fluorescence simulations matched the empirically determined input rates of the simulations.

We modeled the species of free fluorescent label B^*, bound fluorescent label LB^*, bleached label B, bleached bound label LB and free ligand L according to the fundamental rate equation system of Eq. 4 in a glass capillary, see Fig.1 b. The geometry comprised a glass capillary (ID 300 µm, OD 400 µm), placed on a silicon block of 400 µm thickness. The cover slip was 200 µm thick and was placed on top of the capillary. The space outside the capillary between silicon block and glass cover slip was filled with immersion oil. The heating IR laser heated with a gaussian beam profile (minimal width 12 µm, NA=0.12, power density 100 W/m, attenuation length 400 µm in water, no absorbance in glass/silicon, z-focus height 200 µm above the capillary center) and the LED homogeneously illuminates the capillary on a length of 400 µm.

The fluorescence parameters of the simulated 12mer in 0.1xPBS at 19°C were determined to be

$$\begin{aligned}
F_{\text{Free}} &= 1 \\
F_{\text{Bound}} &= 0.9 \\
\frac{\partial F_{\text{Free}}}{\partial T} = \frac{\partial F_{Bound}}{\partial T} &= -0.026\text{K}^{-1} \\
k_{\text{Bleach,free}} &= 0.0021\text{s}^{-1} \\
k_{\text{Bleach,bound}} &= 0.0029\text{s}^{-1} \\
k_{\text{off,offset}} &= 117.890 \\
k_{\text{off,slope}} &= -36313.5\text{K} \\
k_{\text{on}} &= 3.5 \cdot 10^{4}\text{M}^{-1}\text{s}^{-1} \\
B^*_{\text{tot}} &= 2\text{nM} \\
L_{\text{tot}} &= 0\text{M} \wedge 2.5\mu\text{M} \\
D &= 1.5 \cdot 10^{-10}\text{m}^2\text{s}^{-1} \\
D_{\text{T}} &= 1.8 \cdot 10^{-12}\text{m}^2\text{s}^{-1}\text{K}^{-1}
\end{aligned}$$

with the temperature-dependent off-rate

$$k_{\text{off}}(1/T) = \exp\left(k_{\text{off,offset}} + k_{\text{off,slope}} \cdot \frac{1}{T}\right) \cdot \text{s}^{-1}$$

Note that k_{off} used in the simulation from the k_{off}-fit was not equal to the measured k_{off} of 12mer at 19°C (k_{off}=0.017 s^{-1}) of SI-Tab. 3. The simulation yielded for fluorescence intensities, which were analyzed in the same way as the measured ones. The analysis of the simulated fluorescence intensities yielded for values matching the experimental data (for fixed K_d=47 nM in equilibrium).

The volume force on the fluid took light pressure and static fluid pressure into account. The simulation was conducted similarly to the experiment with 50 seconds pre heat phase, 40 s heat phase and 60 s post heat phase. The simulation started in kinetic equilibrium according to the binding curve Eq. 1. The simulation yielded kinetic rates which are in good agreement with the rates of the experiment:

$$\begin{aligned}
k_{\text{off,simulation result}} &= 0.0016\ \text{s}^{-1} \\
k_{\text{on,simulation result}} &= 3.45 \cdot 10^4\ \text{M}^{-1}\text{s}^{-1}
\end{aligned}$$

6 Independence of kinetic rates on label site

We wanted to test, if kinetics could be still detected in the fluorescence signal, if the hybridization of the strands was not in the local environment of the label (label attached to a hybridizing base) but in a more distant location from the hybridizing base pairs, see SI-Fig.5. In all measurements, the fluorescent label was attached to the 5'-end of the 16mer strand to which the (shorter) complementary DNA strand could bind. We measured similar kinetic rates for a system of a fully complementary 12mer strand that started hybridization at the 3'end (opposite) end the labeled strand, leaving a distance of 4bp=(16-12)bp between the label and the hybridized base pairs, see SI-Tab. 1. The measured K_d and kinetic rates of this system were similar to the results of the system which started hybridization at the labeled 5'end. We concluded that the change in the electronic configuration due to (distant) hybridization was sufficient to change the quantum yield of the fluorophore, also reported by Bielec[5] et al and KMST was capable to detect kinetics due to a (conformational) change of the local environment of the label.

Kinetic rates distant fluorescent label							
		Salt 0.1xPBS					
1/T [1000/K]	Temp[°C]	K_d [nM]	Std.Dev	koff [1/s]	Std.Dev	kon [1/Ms]	Std.Dev
3.46	16	1.3E+1	5.0E+0	3.6E-4	2.0E-4	2.8E+4	9.0E+3
3.39	22	1.8E+2	2.0E+1	5.7E-3	1.0E-3	3.2E+4	5.3E+3
3.35	25	1.1E+3	2.7E+2	3.9E-2	1.0E-2	3.5E+4	3.0E+3

SI-Table 1: K_d, off-rate and on-rate with deviations for DNA hybridization measurement of a distantly attaches fluorophore are similar to strands with closely attached fluorophores compared to SI-Tab.3-5.

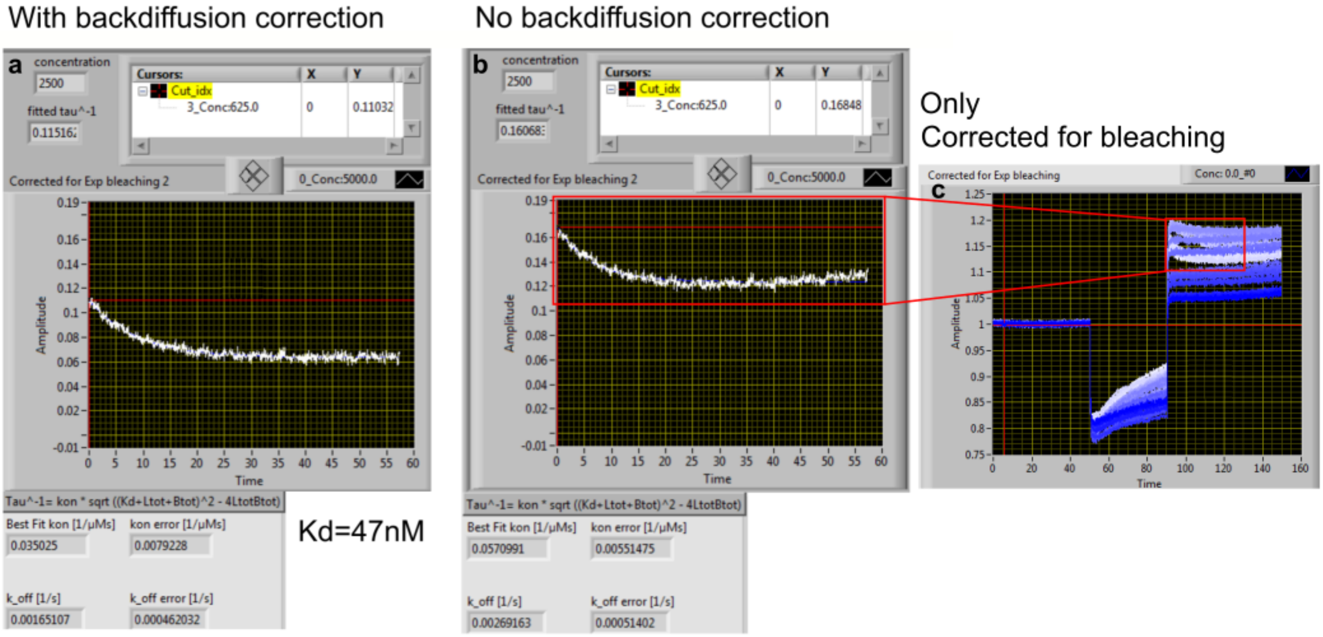

SI-Figure 2: ***Backdiffusion contribution to kinetic analysis a*** *Bleaching- and backdiffusion-corrected fluorescence intensity of a 12mer at 19°C for 2.5µM ligand and 2nM binder with resulting on- and off-rate analyzed as described in SI-4.* ***b*** *Analysis of the same fluorescence intensity without backdiffusion correction, that would have been the division by the averaged zero-ligand intensity (L_{tot}= 0, darkest blue in* ***c****), and resulting kinetic relaxation time constant and rates.* ***c*** *Fluorescence intensities of a dilution series (triplicates per concentration) with only bleaching correction but no diffusion correction.*

7 Influence of diffusion on fluorescence analysis

To test the accuracy of the analysis of the kinetic rates with regard to the deconvolution of the diffusion contribution, we performed the analysis of 12mer at 19°C with and without diffusion correction, see SI-Fig.2. This corresponded to the scenario that the ligand was diffusing but the labeled binder would not diffuse. We found that, when the correction for backdiffusion was dropped, the resulting rates changed by a factor less than two. With regard to the orders of magnitude, by which the measured kinetic rates differ within literature, the factor smaller two is comparably small. We took this finding as an indicator that the analysis method showed robust results regarding differences of diffusion properties of the binder and ligand.

Next, we used our 3D-Comsol simulations to test, how the fluorescence intensities would look and how the kinetic rates would be fitted, if one of the reactants exhibited strongly different diffusion coefficient, while the on-rate and off-rate did not change. Therefore, we simulated two cases: In the first, the ligand had tenfold increased diffusion coefficient D_L=D_{LB}=10xD_B, i.e. because the non-labeled ligand was much larger than the labeled binder. In the second case, the labeled binder had tenfold increased D_B=D_{LB}=10xD_L. In both cases, we assumed that the diffusion coefficient was the same for bound ligand LB and the respective larger free L or B, which had the higher diffusion coefficient. The diffusion behavior of the larger reactant would not change, hen bound to the much smaller reactant.

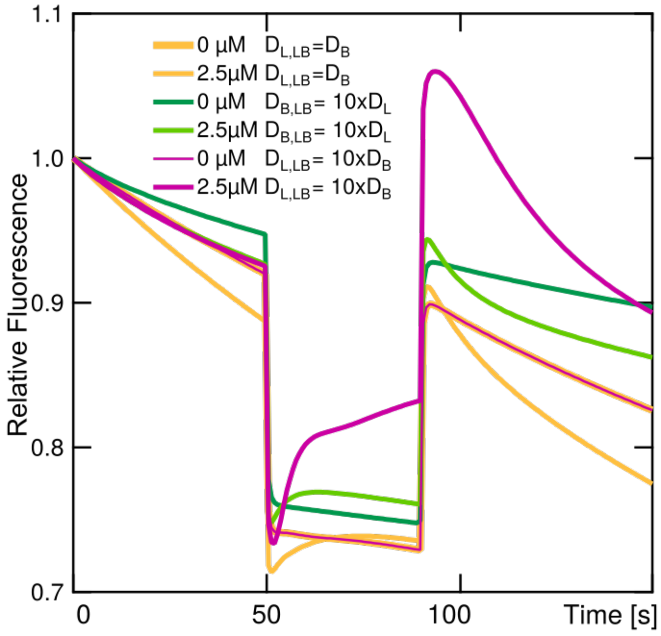

SI-Figure 3: ***Simulated fluorescence for various diffusion behavior*** *Simluated fluorescence for similar diffusion behavior (yellow), larger fluorescent binder (green) and larger fluorescent ligand (purple) for 0nM and 2500nM ligand concentration with the rates of 12mer at 19°C. The fluorescence intensity of the larger fluorescent binder simulations were similar to the results of equal diffusion behavior, with similar kinetic rates. For the larger ligand simulations, the labeled binder accumulated in the top center of the capillary, see SI-Fig.4 g and fluorescence restored quickly. The analyzed kinetic rates differ by a factor of 5 in comparison with the similar diffusion behavior simulations.*

For the first case, we found that the fluorescence for high ligand concentration L_{tot}=2,5µM (over 1000-fold excess of binder B_{tot}=2 nM) looked significantly different for the hot phase (50-90 seconds), see purple lines SI-Fig.3. The deviation of the simulated detected fluorescence at the beginning of the hot phase, compare SI-Fig.4 a and e, can be explained by the concentrations of free L, free B* and bound LB*: During the heating time, the absolute fluorescence initially dropped due to the quick temperature change. Shortly after the temperature change, within about 10 seconds, bound complexes diffused quickly (D_{LB}=10xD_B) from the cold non-illuminated region (c_{LB^*}>c_{B^*}) back into the top center of the capillary (heated area, illuminated), see SI-Fig.4 b and f, and the fluorescence increased quickly again. In the hot center, the bound complexes unbound, see SI-Fig.4 c and g, the fast diffusing unbound ligand molecules moved away from the top center of the capillary, see SI-

Fig.4 d and h, whereas the slowly diffusing labeled binder molecules got stuck at the top center. This left an excess of unbound labeled binder molecules in the top center of the capillary, see SI-Fig.4 c, and explained the increased fluorescence (purple) in the hot phase.

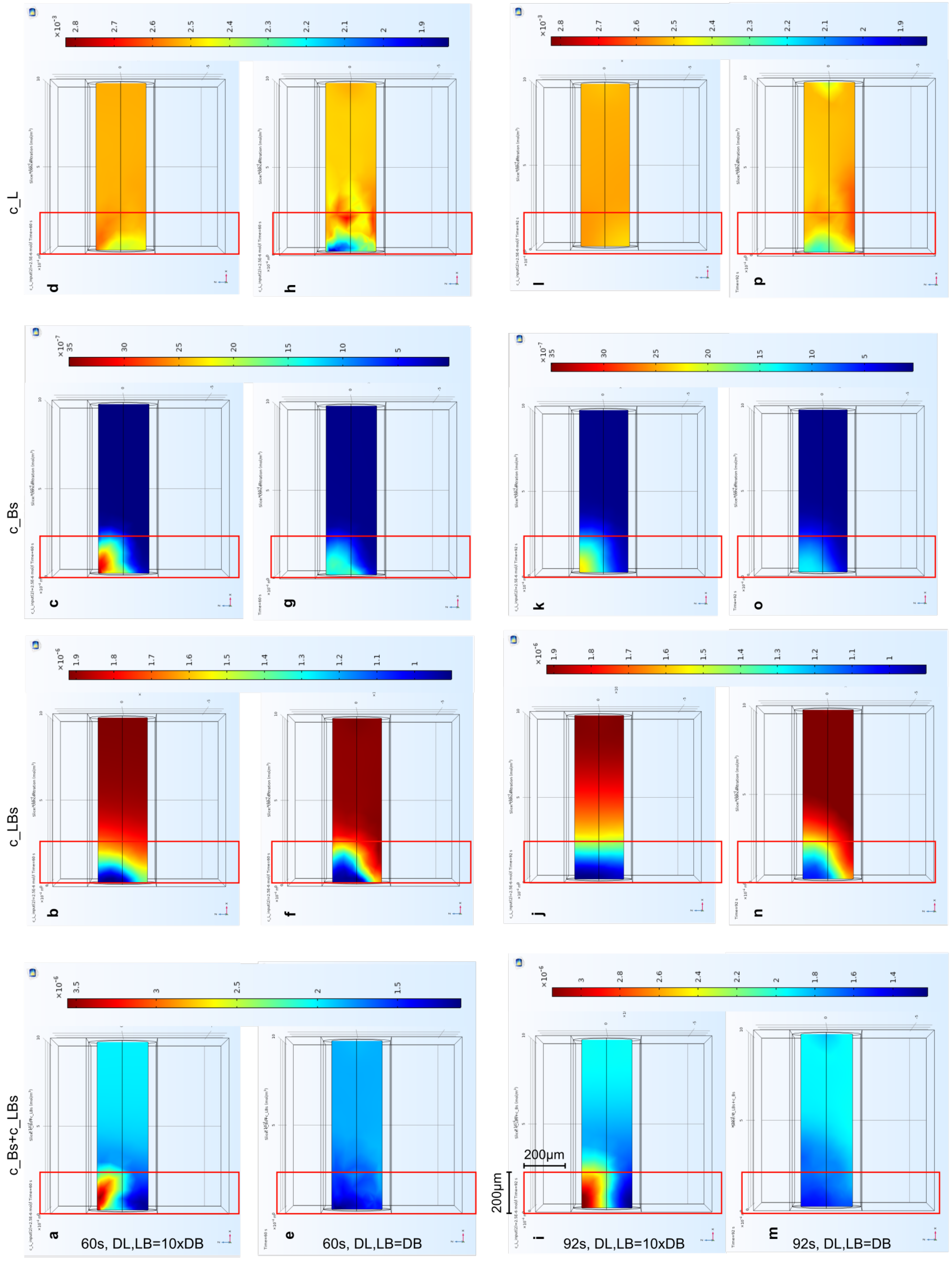

SI-Figure 4: Simulated concentrations of free L, free B* and bound LB* a-h *at 60 seconds during the hot phase and* ***i-p*** *at 92 seconds shortly after the rebinding start for* L_{tot}*=2.5µM and* B^*_{tot}*=2nM.* ***A-d*** *and* ***i-l*** *show the concentrations for tenfold increased ligand diffusion coefficient* $D_{LB^*}=D_L=10xD_B$*. e-h and m-p show the concentrations for equal ligand diffusion coefficient* $D_{LB^*}=D_L=D_B$*. The illuminated region (200µm width) is shown by the red rectangle and shows the symmetrical half of the simulated capillary. The laser focus is on the left edge of each plot.*

When the laser was switched off, the initial fluorescence level was higher due to accumulated free labeled binders in the top center capillary, see SI-Fig.3 purple line at 90 seconds and SI-Fig.4 i and m. The kinetic relaxation of rebinding free labeled binder, see SI-Fig.4 k, and free ligand see SI-Fig.4 l, yielded for slower kinetics. The analysis of the simulated fluorescence intensities yielded for an on-rate $7x10^3 M^{-1}s^{-1}$, which was about one fifth of the input rate $3.5x10^4 M^{-1}s^{-1}$. The analysis of the off-rate yielded for $3.3x10^{-4} s^{-1}$ which was also about one fifth smaller than the input rate. This deviation of the analyzed kinetics from the simulations with equal diffusion behavior could be explained by the reduced homogeneously distributed free binders. In the analysis, we assumed homogeneously distributed binder and ligand in the detected volume, see SI-2. But due to non-homogeneous accumulation of labeled binder in the top center, the assumption of homogeneity was not as valid as in the similar diffusion behavior simulations, see SI-Fig.4 k and l, which were less homogeneously distributed than SI-Fig.4 o and p. In the second case, the fluorescent binder had tenfold increased diffusion coefficient, e.g. a small compound that binds to a larger (labeled) protein. The simulated fluorescence showed less bleaching and similar behavior during the heating period and post-heating period, resulting in a fitted on-rate of $5.6x10^4 M^{-1}s^{-1}$ and off-rate of $2.7x10^{-3}s^{-1}$. The on-rate was a factor of 1.6 higher and the off-rate a factor of 1.6 smaller than the input parameters, which is very close to the input parameters. K_d stayed the same, as we only varied diffusion behavior and not binding behavior.

We interpret the variation of analyzed kinetic rates by smaller than five-fold for dissimilar diffusion behavior of the reactants as a support of our claim that our applied analysis is robust against different sizes of ligand and binder. With regard to the magnitudes of differently reported kinetic rates in literature our reported variations were comparably small. Taken together, the simulation results suggest that if the label could be attached to either the binder or the ligand, the larger molecule should be labeled to reduce systematic errors within the kinetic rate analysis.

8 Optimal conditions for KMST measurements

We could characterize four conditions for optimal applicability of KMST. First, for systems with small dissociation constants K_d < 1 nM, the labeled binder concentration was required to be B^*_{tot} < 1 nM (better in 100 pM range) to allow useful fitting of Eq. 1. But B^*_{tot} < 1 nM led to too low absolute fluorescence signals (<2000 counts) in the detector (recommended >10000). The fluorescence signals were more difficult to analyze due to a smaller signal-to-noise ratio, decreased relative jump heights and too small $F_{kinetic,amplitude}$. This limited the determination of kinetic rates to systems with K_d > 1 nM. In order to increase the absolute fluorescence counts, increasing the LED excitation intensity did not solve the problem, as then bleaching was so strongly pronounced, that triplicate measurements of a single sample capillary were not useful, due to almost completely bleached samples after the first measurement. Also, for strong bleaching, the fluorescence was strongly dominated by the bleaching component, making it difficult to distinguish the kinetic contribution from bleaching.

Second, the detection of fast kinetic relaxation of $\tau_{kinetic}^{-1} > 1s^{-1}$, e.g. for high ligand concentrations and for high on-rates, was limited by the heating, cooling and fluorescence detection time scale $\tau_{cooling}^{-1} \approx 5s^{-1}$.This was problematic for two reasons. First, the superposition of fluorescence change due to heating and kinetic relaxation became more difficult to dissect, as they were on the same timescale. Second, the assumption of an immediate temperature jump and valid applicability of Eq.2 was only valid for $\tau_{kinetic}^{-1} \ll \tau_{cooling}^{-1}$. To unravel kinetics of systems with faster equilibration, the analysis would need to to incorporate time-dependent temperature equilibration with possibly the lack of an analytic description of the time constant.

Third, the KMST measurements depended on temperature-dependent binding and unbinding of biological complexes. This required a significant enthalpic contribution ΔH^0 which led to temperature-dependent K_d. Biological complexes with very weak enthalpic contribution $\Delta H \approx 0$ would not change binding in the heating phase and no kinetic recombination in the post heating phase would be detected. Measurements with p38-α MapKinase with BIRB, SB203580, SB239063 (not shown) did not yield for fingerprints in the fluorescence intensities, most likely due to an insufficient enthalpic contribution.

Fourth, the absolute fluorescence change upon binding required to be significantly large. This corresponds to a significant change of the quantum yield upon binding, that is a large difference of $\hat{F}_{Free} - \hat{F}_{Bound}$ in Eq.7. In the analysis, to detect the kinetic relaxation fingerprint, a sufficient high value of $F_{kinetic,amplitude}$ > 0.05 was crucial. The company Nanotemper, which commercializes MST, has developed in the past dyes with ever increasing thermal binding signal, so that also in KMST, the sensitivity to measure kinetics will increase over time. The origin of the binding-dependent fluorescence intensity may play a minor role, e.g. it may stem from a change in conformation of the complex upon binding that leads to a change in the electronic configuration of the fluorophore. Thus, the fluorophore does not necessarily need to be in close proximity to the binding site, reducing the label influence on binding characteristics.

Additionally, KMST experiments require the system to be at kinetic equilibrium in the pre-heating phase. Therefore it has to be ensured that the sample has reached equilibrium after mixing the reactants[6] and incubating for 20 minutes. The measured relaxation time constants were in the order of 10 seconds. We draw the conclusion that the sample had equilibrated during the 20 minute incubation after capillary filling and thus the system was in equilibrium in the pre-heat phase.

9 DNA samples & preparation

Each dilution series comprised 16 vials, in a 1:1 dilution between the vials. In vial 15 and 16 only binder with no ligand was used for the determination of $F_{diffusion}$. The binder concentration was set below K_d to yield for binding curves to obtain reasonable K_d values and correctly apply Eq.1. The labeled binder concentrations were 2nM to yield for good K_d fits as well as sufficient fluorescence counts. The range of the ligand concentration was chosen to be symmetric around K_d, to obtain a valid binding curve, reaching the unbound plateau and bound plateau for the fraction bound curve, see Fig3 b.

The DNA strands used for hybridization kinetics determination were purchased from biomers (Ulm, Germany). The fluorescent labeled binder was a 16mer sequence Cy5-5'CCT CAT CCA TAG TTG C3' and the complementary ligands were

10mer: 5'a tgg atg agg3'

12mer: 5'cta tgg atg agg3' or 5'gca act atg gat3' (distant from label, see SI-6)

14mer: 5'aa cta tgg atg agg3'

16mer: 5'g caa cta tgg atg agg3', see SI-Fig.5.

The ligand strands bound to the binder strands starting at the Cy-5-End to obtain a strong binding-dependent change of the fluorescence signal. All used strands were factory HPLC purified before purchase. The stock concentration for all strands was 100µM dissolved in water. As the fluorophore was located next to the hybridizing nucleotides, the fluorescence became binding-dependent, similarly reported earlier.[7] Both strands were not self-complementary and no side reactions were expected.

The DNA strands were dissolved in 0.75x, 0.5x, 0.25x and 0.1x PBS buffer (stock: 10xPBS invitrogen ThermoFisher, diluted in nuclease-free water H_2O, Ambion). To avoid sticking of material to the capillary walls, 0.05% (wt/vol) Tween 20 (NanoTemper) was added to all buffers. The experiment ambient temperatures were chosen to be below the melting temperature T_m of the fully complementary strand to obtain a high change of fraction bound due to the temperature change. SI-Fig.6 a shows the raw fluorescence unit of the melting curve of 12mer in 0.1xPBS (orange), an empty vial (background, blue) and fluorescent binder and Eva green dye only (green). The concentration of binder and ligand were 10µM each for the melting curve and 10µM for the dye and binder only curve. To correct for the temperature dependence of the dye, we subtracted the background from the melting curve and the dye and binder-only curve, respectively, and then divided the melting curve by the dye and binder-only curve to obtain the fraction bound, see SI-Fig.6 b. The melting curve was recorded with a Biorad Thermocycler C 1000 between 5°C and 80°C. We found T_m=43°C and concluded that the strands were bound in the pre heat and post heat phase and heating the sample with the infrared laser melted the hybridized DNA strands. When the laser was switched off, the DNA strands hybridized and kinetics were detected. Simulations of the 12mer (10µM like melting curve) with NUPACK (0.05M Na^+) yielded for T_m=38°C. Simulation of T_m for the used strands (2nM binder) under the respective Na^+ conditions in PBS buffer were rather inconclusive for low salt concentrations and short strands. The simulation of melting temperature with Oligo Calc[8] (salt adjusted and nearest neighbor mode) and NUPACK[9] are shown in SI-Tab. 2.

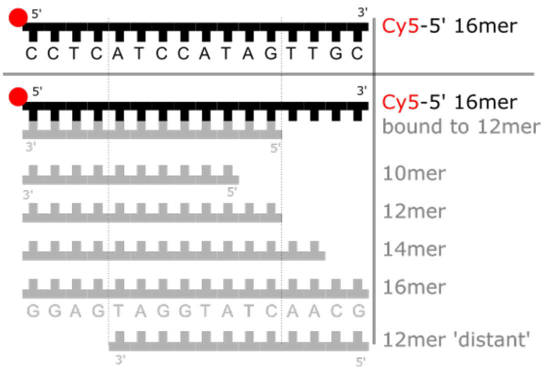

SI-Figure 5: DNA strands used for hybridization measurements *The 16mer (binder) with a Cy5-label (red circle) at the 5'end was used for all experiments. To the Cy5-labeled 16mer, the (non-labeled) complementary 10mer, 12mer, 14mer, 16mer (ligand) could bind. The 12mer 'distant from label' was used to test if binding could be detected, if the binding site (start of hybridizing strand) was in a distance of 4 base pairs from the Cy5 label.*

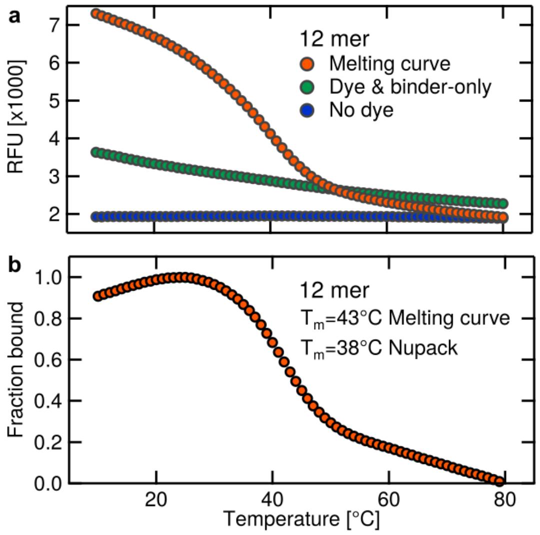

SI-Figure 6: Melting curve and fraction bound a *shows the raw fluorescence units of 1xEva Green intercalating dye obtained with a Biorad Thermocycler of the complementary 12mer in 0.1xPBS (orange), binder and dye only (green) and background (blue). The concentration of the strands were 10µM for the melting curve, each, and 10µM for the binder-only curve.* ***b*** *shows the fraction bound, which was obtained by temperature correction of the dye. Therefore, the background was subtracted from the melting curve and the binder-only curve, respectively, and then the melting curve was divided by the binder-only curve. The fraction bound was normalized to one. T_m was 43°C.*

SI-Table 2: Overview of calculated melting temperatures for used DNA strands

T_m [°C] calculated for x PBS	0.1	0.25	0.5	0.75
12 mer NUPACK	-	-	23.5	25
12 mer Oligo Calc Nearest Neighbor	8.1	14.9	19.9	22.8
12 mer Oligo Calc salt adjusted	26.7	33.3	38.2	41.2

T_m [°C] calc. for strand lengths 0.1 x PBS	10	12	14	16
Oligo Calc nearest neighbor	-2.1	8.1	16.2	25.9
Oligo Calc salt adjusted	20.3	26.3	28.2	38.4

Results and Discussion

10 Summarized measured kinetic rates and dissociation constant

SI-Tables 3 – 5 show the corresponding values of the measured dissociated constant K_d in M, k_{off} in s^{-1} and k_{on} in $M^{-1}s^{-1}$ with respective standard deviations of Fig.4 & 5 in the article. We also tried to measure the kinetic rates of the used 12mer by a quick temperature jump experiment with a thermocycler of Biorad with added EvaGreen intercalating dye. The heating characteristic was about 10 seconds for a jump from 70°C to 10°C and the technique was used earlier to obtain kinetic relaxation time constants of DNA 51mers with FRET.[10] Analysis of the EvaGreen signal did not yield for a valid kinetic relaxation signal. This may stem from difficulties associated with the measurement of kinetics with Eva Green dye as well as the readout timing of the machine. Also, the temperature characteristic may have been not quick enough to extract kinetics.

Equilibrium constant K_d									
		Salt [xPBS]							
		0.1		0.25		0.5		0.75	
1/T [1000/K]	Temp[°C]	Kd [M]	Std.Dev	Kd [M]	Std.Dev	Kd [M]	Std.Dev	Kd [M]	Std.Dev
3.53	10								
3.49	13	1.9E-8	2.6E-9						
3.46	16	1.1E-8	9.1E-10						
3.42	19	4.7E-8	7.8E-9						
3.39	22	1.6E-7	1.5E-8	1.4E-8	4.4E-9			3.9E-10	2.1E-10
3.35	25	1.5E-6	4.9E-7	5.3E-8	1.8E-8	4.7E-9	3.2E-9	6.7E-10	4.4E-10
3.32	28					1.2E-8	1.1E-8	1.0E-9	6.5E-10
		Length [#bp] in 0.1xPBS							
		10		12		14		16	
		Kd [M]	Std.Dev	Kd [M]	Std.Dev	Kd [M]	Std.Dev	Kd [M]	Std.Dev
3.53	10	4.5E-9	2.1E-9						
3.49	13	4.3E-8	1.7E-8	1.9E-8	2.6E-9				
3.46	16	1.2E-7	3.2E-8	1.1E-8	9.1E-10				
3.42	19	3.8E-7	1.9E-7	4.7E-8	7.8E-9				
3.39	22	3.9E-6	9.6E-7	1.6E-7	1.5E-8	2.8E-9	7.2E-10	1.1E-9	2.3E-10
3.35	25			1.5E-6	4.9E-7	1.1E-8	5.6E-9	7.6E-9	1.7E-9
3.32	28					5.1E-8	1.9E-8	9.4E-9	1.9E-9

SI-Table 3: Dissociation constant K_d in Molar and standard deviation in Molar for DNA hybridization measurements.

Off-rate k_{off}									
		Salt [xPBS]							
		0.1		0.25		0.5		0.75	
1/T [1000/K]	Temp[°C]	koff [1/s]	Std.Dev	koff [1/s]	Std.Dev	koff [1/s]	Std.Dev	koff [1/s]	Std.Dev
3.53	10								
3.49	13	3.7E-4	7.5E-5						
3.46	16	2.4E-4	3.0E-5						
3.42	19	1.7E-3	4.6E-4						
3.39	22	7.8E-3	1.2E-3	2.9E-3	9.7E-4			4.2E-4	2.4E-4
3.35	25	3.3E-2	1.6E-2	1.1E-2	4.1E-3	5.2E-3	4.2E-3	9.8E-4	6.5E-4
3.32	28					1.3E-2	1.5E-2	1.6E-3	1.0E-3
		Length [#bp] in 0.1xPBS							
		10		12		14		16	
		koff [1/s]	Std.Dev	koff [1/s]	Std.Dev	koff [1/s]	Std.Dev	koff [1/s]	Std.Dev
3.53	10	1.2E-4	5.6E-5						
3.49	13	7.6E-4	3.2E-4	3.7E-4	7.5E-5				
3.46	16	3.9E-3	1.1E-3	2.4E-4	3.0E-5				
3.42	19	1.7E-2	8.6E-3	1.7E-3	4.6E-4				
3.39	22	4.2E-2	1.6E-2	7.8E-3	1.2E-3	2.3E-4	6.1E-5	5.3E-5	1.1E-5
3.35	25			3.3E-2	1.6E-2	7.5E-4	3.8E-4	3.9E-4	8.6E-5
3.32	28					3.6E-3	1.5E-3	4.8E-4	1.2E-4

SI-Table 4: Off-rate k_{off} in s^{-1} and standard deviation in s^{-1} for DNA hybridization measurements.

On-rate k_{on}									
		Salt [xPBS]							
		0.1		0.25		0.5		0.75	
1/T [1000/K]	Temp[°C]	kon [1/Ms]	Std.Dev	kon [1/Ms]	Std.Dev	kon [1/Ms]	Std.Dev	kon [1/Ms]	Std.Dev
3.53	10								
3.49	13	2.0E+4	2.8E+3						
3.46	16	2.2E+4	2.0E+3						
3.42	19	3.5E+4	7.9E+3						
3.39	22	4.8E+4	6.1E+3	2.2E+5	1.1E+4			1.1E+6	2.4E+5
3.35	25	2.2E+4	8.2E+3	2.1E+5	3.1E+4	1.1E+6	4.5E+5	1.5E+6	1.1E+5
3.32	28					1.1E+6	6.5E+5	1.6E+6	1.4E+5
		Length [#bp] in 0.1xPBS							
		10		12		14		16	
		kon [1/Ms]	Std.Dev	kon [1/Ms]	Std.Dev	kon [1/Ms]	Std.Dev	kon [1/Ms]	Std.Dev
3.53	10	2.6E+4	1.1E+3						
3.49	13	1.8E+4	1.6E+3	2.0E+4	2.8E+3				
3.46	16	3.3E+4	1.2E+3	2.2E+4	2.0E+3				
3.42	19	4.6E+4	2.7E+3	3.5E+4	7.9E+3				
3.39	22	1.1E+4	3.3E+3	4.8E+4	6.1E+3	8.4E+4	3.3E+3	4.8E+4	2.9E+3
3.35	25			2.2E+4	8.2E+3	6.6E+4	9.4E+3	5.1E+4	1.7E+3
3.32	28					7.0E+4	1.6E+4	5.2E+4	6.9E+3

SI-Table 5: On-rate k_{on} in $M^{-1}s^{-1}$ and standard deviation in $M^{-1}s^{-1}$ for DNA hybridization measurements.

11 Van 't Hoff analysis

Table 1 in the article shows the fitted Van 't Hoff parameters $\Delta H^0, \Delta S^0, \Delta G^0$, from the K_d plot over inverse temperature of Fig.5 e & f. We compared the values from the KMST measurements with values from the melting curve. To extract K_d over inverse temperature from the linear regime of the melting curve (between 3.05 and 3.3x10^{-3} K^{-1}), we applied the fraction of bound complex P_{bound} and $K_d = \frac{L \cdot B^*}{LB^*}$. SI-Fig.7 shows the K_d values and Van 't Hoff fits for the KMST data and the K_d obtained from the melting curve. The thermodynamic values from the Van 't Hoff plot of the K_d from the melting curve yielded for ΔH^0=-47±1 kcal mol^{-1} and ΔS^0=-117±1 kcal K^{-1} mol^{-1}. The temperature dependence was similar for both measurement methods (KMST: ΔH^0=-58 kcal mol^{-1}) but the entropies deviated (KMST: ΔS^0=-168±56 kcal K^{-1} mol^{-1}). The deviation of the K_d from the melting curve and KMST could be explained as follows: First, the Eva Green intercalating dye may be interfering with the Cy5-label of the binder in a way that the melting curve signal and thus did not properly report the fraction of bound complexes. Second, the quantification of K_d by the melting curve is most suitable for temperatures around T_m=43°C. The extraction of K_d data from the melting curve well below T_m is difficult because the intercalation fluorescence signal does not change significantly at T<<T_m, as almost all strands are hybridized. As all KMST measurements were carried out at T<<T_m, direct comparison is difficult. Third, to obtain a good signal from melting curves, the concentration of both strands needed to be in the µM range. This range was 1000-fold higher than for the KMST measurements (2 nM). The high concentration of strands may also shift the melting temperature signal to higher temperatures.

We also tried to measure the K_d and kinetic rates with Dynamic Light Scattering (DynaPro NanoStar, Wyatt) but could not detect the probes (10µM sample concentration). Due to the small size and low concentration, the correlation function of the labeled binder could not be distinguished from the buffer-only correlation function.

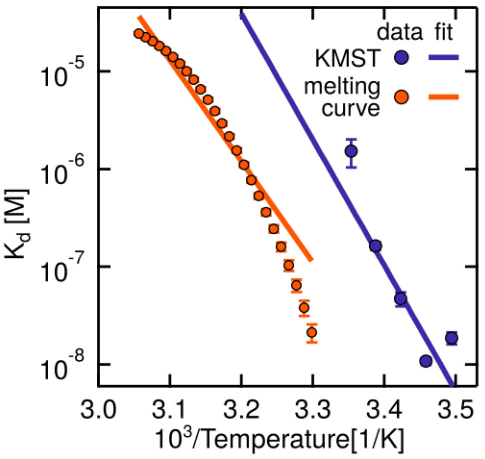

SI-Figure 7: Comparison of Van 't Hoff fits from melting curve and KMST experiments *K_d and Van 't Hoff fits of a complementary 12mer in 0.1xPBS which was obtained by melting curve with Eva Green dye (orange, from SI-Fig.6 b) and KMST measurements. Both curves show similar slopes ΔH^0=-47±1 kcal mol^{-1} (melting curve) and ΔH^0=-58±16 kcal mol^{-1} (KMST) but dissimilar ΔS^0=-117±1 kcal K^{-1} mol^{-1} (melting curve) and ΔS^0=-168±56 kcal K^{-1} mol^{-1} (KMST). The shift of the measured K_d may stem from interferences of the intercalation dye with the probe. Furthermore, the applicability of melting curves (see SI-5) to obtain K_d values well below T_m is limited.*

12 Thermodynamic analysis

The identification of the temperature dependent kinetic rates by the Eyring-Polanyi equation allows for determination of thermodynamic quantities. Although, the connection of kinetic quantities with thermodynamic quantities depends on details of transition states[11] also for short oligomer hybridization of DNA, it is possible within limits.[12,13] Following the works of Dupuis et al.[12] the Eyring equation connects thermodynamic quantities of the binder-ligand system with the kinetic rates

$$\ln(k/(\nu \cdot k^0)) = \frac{-\Delta H^\ddagger}{RT} + \frac{\Delta S^\ddagger}{R} \tag{8}$$

With k the on-rate or off-rate, $\nu$the attempt frequency, which is implicitly temperature dependent, k^0 equals unity for the off-rate and M^{-1} for the on rate, $\Delta H^\ddagger{}_{on}$ the association or $\Delta H^\ddagger{}_{off}$ dissociation enthalpy and $\Delta S^\ddagger{}_{on}$ the association or $\Delta S^\ddagger{}_{off}$ dissociation entropy. The chosen identification of Eq. 8 leads to identical Eyring fits and Arrhenius fits. The thermodynamic quantities of the Eyring fits are shown in SI-Tab. 6 for hybridization and SI-Tab. 7 for dissociation. Note that the Arrhenius activation energy $E_{A,on}$ is identical to $\Delta H^\ddagger_{on}$ for the on-rate and $E_{A,off}$ is identical to $\Delta H^\ddagger_{off}$ for the off-rate, respectively.

The attempt frequency $\nu = 4s^{-1}$ was determined similarly to previous works of Dupuis et al.[12] whose analysis we closely follow in this section. Knowledge of the attempt frequency allows for connection of kinetic quantities with thermodynamic quantities by interpreting the pre-exponential factor of the Arrhenius equation with the entropy term of the Eyring-Polanyi equation. TST treats the hybridization of oligomer DNA strands as a one-way crossing over a transition state barrier at the attempt frequency of ν. ν is determined by diffusion limited duplex formation, which is calculated $k_{diff} = 4\pi \cdot R_{DNA} \cdot D \cdot 1000 \cdot N_A$and has unit M^{-1}s^{-1}. The DNA radius is estimated to be 12bp times 3Å/bp and the diffusion coefficient is 1x10^{-10} m^{2}s^{-1} and N_A the Avogadro Number 6.02x10^{23} mol^{-1}. The binder DNA concentration was 2 nM. We yield for k_{diff}=2x10^9 M^{-1}s^{-1}. To appropriately treat the diffusion constant, it is corrected by the factors T/295K and 1000/(24x10$^{(247K/(T-140K))}$).[14] The factor 1000 enters because of the conversion to Molar=mol/l=1000mol/m³. Calculating $\nu_{283K} = 3s^{-1}$ and $\nu_{303K} = 5.2s^{-1}$, we chose $\nu = 4s^{-1}$ for all further analysis. The uncertainties of ν do not have an influence on $\Delta H^\ddagger$ and E_A as they only yield for a small and logarithmic dependent shift of $\Delta S^\ddagger$.[12,13] As ν is a fundamental property of the transition state, it is "almost certainly not influenced by salt type or concentration".[13]

In order to compare the thermodynamic quantities' dependencies on salt concentrations and lengths, we plotted the enthalpy and entropy changes for the free, transition and bound state in SI-Fig.8. All changes have been referenced to the bound state. Ideally, $\Delta G^\ddagger{}_{on}$ and $\Delta G^\ddagger{}_{off}$ could be plotted to characterize DNA hybridization as a spontaneous process at the measured conditions. Unfortunately, the error of $\Delta G^\ddagger{}_{on}$ and $\Delta G^\ddagger{}_{off}$ were too large for a concluding remark. Our data suggest, that increasing salt concentration and increasing oligomer length favor the annealing reaction an reduce the enthalpy and entropy barrier from free to bound state.

In 0.1xPBS	$\Delta G^{\ddagger}_{off}$ [a] kcal/mol	$\Delta G^{\ddagger}_{off}$ stdv [b] kcal/mol	$\Delta H^{\ddagger}_{off}$ kcal/mol	$\Delta H^{\ddagger}_{off}$ Stdv kcal/mol	$\Delta S^{\ddagger}_{off}$ cal/K*mol	$\Delta S^{\ddagger}_{off}$ Stdv cal/K*mol	$T\Delta S^{\ddagger}_{off}$ kcal/*mol
10mer	1.0	8.5	80	6	265	20	79
12mer	3.1	26.0	72	18	231	63	69
14mer	4.8	7.6	79	5	249	19	74
16mer	4.9	45.4	49	32	148	108	44
12 mer							
0.1xPBS	3.1	26.0	72	18	231	63	69
0.25xPBS	3.1	42.1	77	30	248	99	74
0.5xPBS	4.6	37.6	55	28	169	84	50
0.75xPBS	4.7	8.3	39	6	115	19	34

SI-Table 6: Thermodynamic parameters for dissociation according to Eyring-plot of the off-rates of Fig.5 c & d. [a]calculated [b]estimated by Gaussian error propagation from fit. Grey values indicate an estimated error of 50% due to lack of data. $T\Delta S$ was calculated for T=298K.

In 0.1xPBS	$\Delta G^{\ddagger}_{on}$ [a] kcal/mol	$\Delta G^{\ddagger}_{on}$ stdv [b] kcal/mol	$\Delta H^{\ddagger}_{on}$ kcal/mol	$\Delta H^{\ddagger}_{on}$ Stdv kcal/mol	$\Delta S^{\ddagger}_{on}$ cal/K*mol	$\Delta S^{\ddagger}_{on}$ Stdv cal/K*mol	$T\Delta S^{\ddagger}_{on}$ kcal/*mol
10mer	-6.0	7.3	8	5	47	18	14
12mer	-6.0	7.1	14	5	67	17	20
14mer	-5.6	5.8	-8	4	-8	14	-2
16mer	-5.3	5.8	3	4	28	14	8
12 mer							
0.1xPBS	-6.0	7.1	14	5	67	17	20
0.25xPBS	-6.3	24.2	-1.5	0.6	16	81	5
0.5xPBS	-7.2	3.1	-1.5	0.75	19	10	6
0.75xPBS	-7.3	5.8	7.6	4	50	14	15

SI-Table 7: Thermodynamic parameters for hybridization according to Eyring-plot of the on-rates of Fig.5 a & b. [a]calculated [b]estimated by Gaussian error propagation from fit. Grey values indicate an estimated error of 50% due to lack of data. $T\Delta S$ was calculated for T=298K.

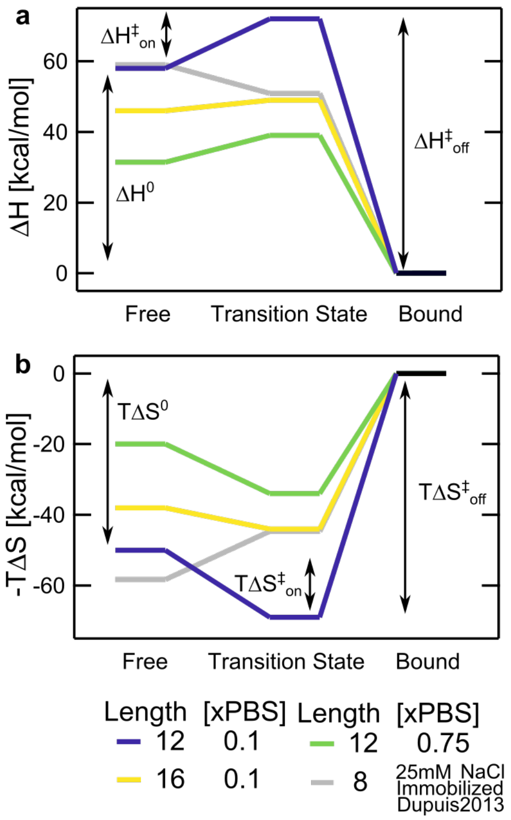

SI-Figure 8: Thermodynamic quantities of DNA hybridization for various oligomer length and salt conditions. **a** the enthalpy and **b** the entropy for free, transition and bound state. Grey data from Dupuis et al.[12]

13 Kinetic rates in crowded solutions

The kinetic rates for crowded solutions with increasing PEG 8000 concentrations are shown in SI-Tab. 8.

Kinetic rates PEG							
		Salt 0.1xPBS					
PEG (w/v%)	Temp[°C]	K_d [nM]	Std.Dev	koff [1/s]	Std.Dev	kon [1/Ms]	Std.Dev
0	16	11	1	0.00024	0.00005	22329	2020
	22	163	15	0.0078	0.001	47933	6114
2.5	16	7.5	2.2	0.00028	0.0001	36848	5255
	22	61	15	0.004	0.001	66762	15347
5.0	16	4.9	0.8	0.00009	0.00003	18474	4513
	22	31	5	0.0012	0.0005	39075	16174
7.5	16						
	22	59	23	0.005	0.002	83729	13299
10.0	16	36	4	0.00036	0.00004	9902	842
	22	263	56	0.007	0.002	26881	3670

SI-Table 8: K_d, off-rate and on-rate with deviations for 12mer DNA hybridization measurement in PEG (w/v%) in 0.1xPBS.

References

[1] C. J. Wienken, P. Baaske, U. Rothbauer, D. Braun, S. Duhr, *Nat. Commun.* **2010**, *1*, DOI 10.1038/ncomms1093.

[2] P. Baaske, C. J. Wienken, P. Reineck, S. Duhr, D. Braun, *Angew. Chemie - Int. Ed.* **2010**, *49*, 2238–2241.

[3] I. Schoen, H. Krammer, D. Braun, *Proc. Natl. Acad. Sci. USA* **2009**, *106*, 21649–21654.

[4] J. F. Eccleston, S. R. Martin, M. J. Schilstra, *Methods Cell Biol.* **2008**, *84*, 445–477.

[5] K. Bielec, G. Bubak, T. Kalwarczyk, R. Holyst, *J. Phys. Chem. B* **2020**, *124*, 1941–1948.

[6] T. H. Scheuermann, S. B. Padrick, K. H. Gardner, C. A. Brautigam, *Anal. Biopchem.* **2016**, *496*, 79–93.

[7] K. Bielec, K. Sozanski, M. Seynen, Z. Dziekan, *Phys.Chem.Chem.Phys* **2019**, *21*, 10798–10807.

[8] W. A. Kibbe, *Nucleic Acids Res.* **2007**, *35*, W43--W46.

[9] J. SantaLucia, D. Hicks, *Annu. Rev. Biophys. Biomol. Struct.* **2004**, *33*, 415–440.

[10] A. Ianeselli, C. B. Mast, D. Braun, *Angew. Chemie - Int. Ed.* **2019**, *58*, 13155–13160.

[11] P. J. Doyle, A. Savara, S. S. Raiman, *React. Kinet. Mech. Catal.* **2020**, *129*, 551–581.

[12] N. F. Dupuis, E. D. Holmstrom, D. J. Nesbitt, *Biophys. J.* **2013**, *105*, 756–766.

[13] K. A. van der Meulen, S. E. Butcher, *Nucleic Acids Res.* **2012**, *40*, 2140–2151.

[14] J. Kestin, M. Sokolov, W. A. Wakeham, *J. Phys. Chem. Ref. Data* **1978**, *7*, 941–948.

Author Contributions

J.A.C.S performed the experiments, the simulations and analyzed the data. J.A.C.S, A.I and D.B. conceived and designed the experiments and simulations, and wrote the manuscript
